# Resolving Geroplasticity to the Balance of Rejuvenins and Geriatrins

**DOI:** 10.14336/AD.2022.0414

**Published:** 2022-12-01

**Authors:** Siamak Tabibzadeh

**Affiliations:** Frontiers in Bioscience Research Institute in Aging and Cancer, Irvine, CA 92618, USA

**Keywords:** geroplasticity, balance, rejuvenins, geriatrins

## Abstract

According to the cell centric hypotheses, the deficits that drive aging occur within cells by age dependent progressive damage to organelles, telomeres, biologic signaling pathways, bioinformational molecules, and by exhaustion of stem cells. Here, we amend these hypotheses and propose an eco-centric model for geroplasticity (aging plasticity including aging reversal). According to this model, youth and aging are plastic and require constant maintenance, and, respectively, engage a host of endogenous rejuvenating (rejuvenins) and gero-inducing [geriatrin] factors. Aging in this model is akin to atrophy that occurs as a result of damage or withdrawal of trophic factors. Rejuvenins maintain and geriatrins adversely impact cellular homeostasis, cell fitness, and proliferation, stem cell pools, damage response and repair. Rejuvenins reduce and geriatrins increase the age-related disorders, inflammatory signaling, and senescence and adjust the epigenetic clock. When viewed through this perspective, aging can be successfully reversed by supplementation with rejuvenins and by reducing the levels of geriatrins.

## Introduction

At the organismal level, aging is evident in all human beings by loss of the ability to reproduce, and dysfunction or loss of function in organs, tissues, and cells (Tables 1-2). There is myriad of cell-centric damages that are associated with aging. In 1981, Harman ascribed aging to be due to a progressive accumulation of temporal changes that lead to an ever-increasing susceptibility to disease and death [1]. According to Hayflick, the common denominator that underlies all modern theories of biological aging is change in molecular structure(s) and, hence, function of cells [2]. This includes damage to bioinformational macromolecules, proteins, carbo-hydrates and lipids as well as concomitant hyperfunction of certain pathways, most notably, of mTOR and NF-κB signaling [3]. Other changes include DNA mutations and genomic instability, glycation, side reactions, and loss of proteostasis (protein folding and proteolysis). Aging is associated with replication-associated DNA damage that perturbs normal cell function as a result of altered specific or global gene expression. changes in transcription and RNA processing modifying protein production. Damage to proteins contributes to cellular aging as a result of formation of mis-folded and aggregated proteins that cause loss of function of proteins and loss of a stable and functional proteome [4-5]. Ever increasing levels of radical oxygen species (ROS) cause an increase in oxidative stressogeneic mutations.

Aging is also associated with mitochondrial DNA damage and dysfunction. As a result of telomere shortening, actively proliferating cells stop their cell division (replicative senescence) by the engagement of p16INK4a/Rb and p19ARF/p53, creating a diseased tissue landscape [6-12]. Changes that contribute to aging also manifest by loss of cellular, tissue and organ function in long lived cells such as neurons and muscle fibers. Occurrence of chronological and replicative damage in stem cells and their loss impairs the tissue regeneration which is essential to the replacement of short-lived cells that are continuously lost with time [13]. Aging causes emergence of inflammation (inflammaging), and increased susceptibility to the development of cancer [14-15].

There is emerging evidence that the cellular changes that occur in aged tissues can be reversed, and aging cells can be rejuvenated. In this paper, the current evidence that supports that aging is plastic (geroplasticity), the conceptual framework of an eco-centric model for this plasticity and data that support for the existence of rejuvenating (rejuvenin) and age inducing (geriatrin) factors that drive such a plasticity are discussed.

**Figure 1.**
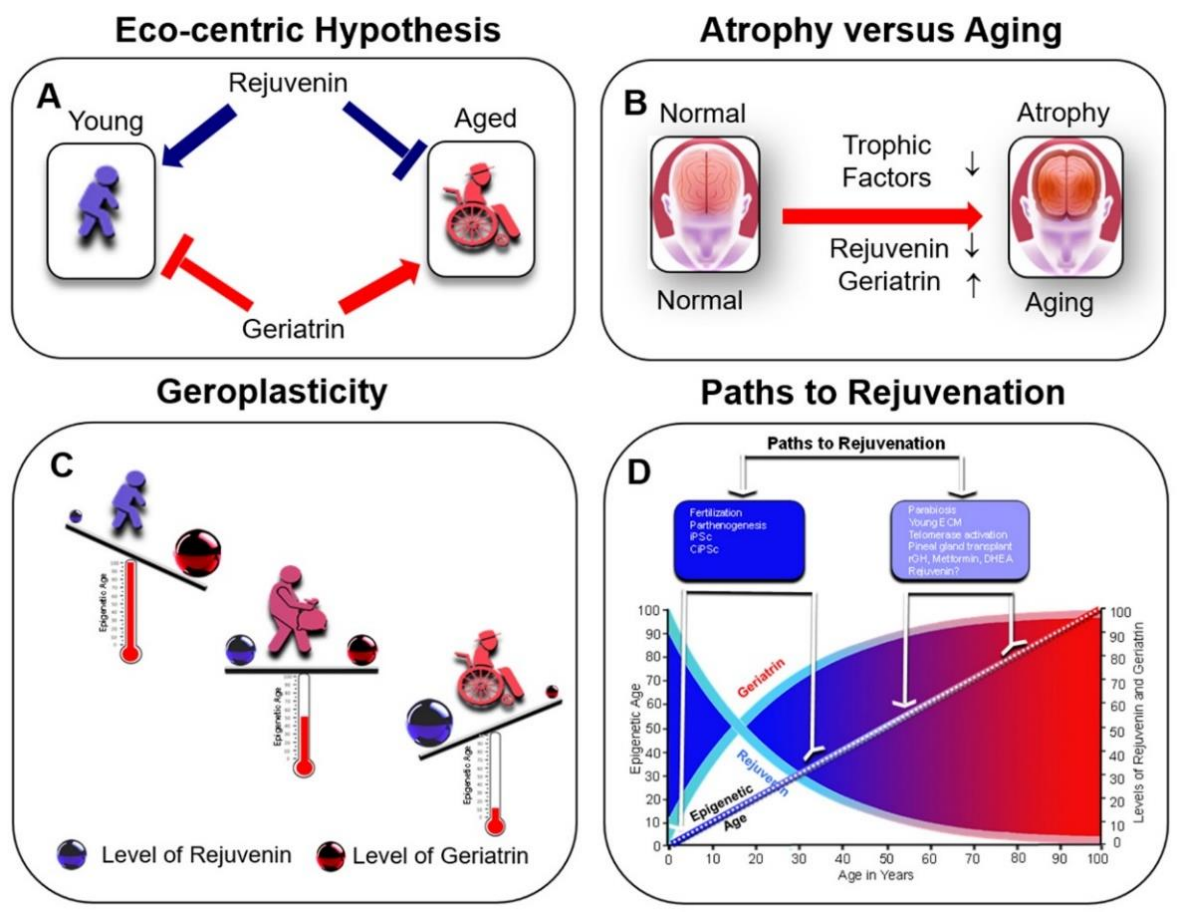
Geroplasticity resolved to the balance of rejuvenins and geriatrins. (A) At the beginning of life rejuvenins provide cell homeostasis whereas, later in life, loss of rejuvenins and escalating levels of geriatrins lead to aging and age-related disorders. (C) Akin to atrophy caused by withdrawal of trophic factors, aging is caused by declined levels of rejuvenins and increased levels of geriatrins. For example, increase in Hcys leads to cortical atrophy (16). (D) Aging leads to an increase in epigenetic age, whereas the diverse rejuvenation strategies drive down the epigenetic age (17-18).

### Eco-centric model of aging

Eco-centric model of aging is founded on the basis of two principles (Fig. 1A-D). First, that homeostasis of organs, tissues, and cells after birth is required to be continuously maintained by an on-going rejuvenation of damaged cells and tissues by a host of rejuvenating factors (rejuvenins) (Fig. 1). A host of age promoting factors (geriatrins) oppose the rejuvenating effects of rejuvenins and induce an aging landscape (Fig. 1). Following production from a host of sources, to be disseminated throughout the body, rejuvenins and geriatrins first enter the interstitium of all tissues and then they transit to peripheral blood, and by passing the Blood-Brain-Barrier (BBB), enter cerebrospinal fluid (CSF) and brain tissues. Second, rejuvenins are abundant early in life, and their age dependent progressive decline with age, leads to the loss of their impact on cell and organ health and a steady increase in epigenetic age (Fig. 1). On the other hand, concomitant with reducing levels of rejuvenins in aging, the levels of geriatrins increase with age exacerbating the effect of rejuvenin loss. This allows for emergence of an onslaught of cell-centric damages that are hallmarks of age-related changes that ultimately manifest at organismal levels (Tables 1-2) [16-18]. If aging is viewed through this lens, treatment of aging becomes feasible by the ensemble substitution of lost rejuvenins and reduction of geriatrins. This strategy, reverses, the age mediated cell mal-functions, preferably resets the epigenetic clock to an earlier time-point, restores homeostasis in cells, tissues and organs and extends health-span and lifespan.

**Table 1:** Impact of aging and senescence on cellular parameters.

Item	Effect	Reference
Integrity of membrane barriers		
Mitochondrial membrane (membrane bound ATP)	↓	19
Nuclear membrane	↓	20
Lysosome membrane	↓	16, 17
Plasma membrane	↓	16, 17
DNA		
Integrity	↓	21
Global DNA methylation	↓	16, 17
Site specific DNA methylation	↑↓	16, 17
Histone modifications		
Global acetylation	↓	22-24
H3K4me3	↑	16, 17
H3K9me	↓	16, 17, 25
H3K9me2	↑↓	25
H3K9me3	↓	26-30
H3K9ac	↑	25, 28, 31-34
H3S10phospho	↑	25, 34
H3K27me3	↑	35-36
H3K56ac	↑	25, 37-38
H4K16ac	↓	16, 17, 25, 37
H4K20me	↓	25
H4K20me2	↓↑	16, 17, 25, 39
H4K20me3	↓↑	39
H3.1	↑	25, 38, 40, 41, 1999
H3.2	↑	25, 38, 40 41
H3.3	↓	40, 41,
H4	↑	25
H2A.1	↑	38, 40, 41
H2A.2	↓	25, 40, 41,
γH_2_AX	↓	25, 42
Chromatin remodeling		
HP1α	↓	16-17
NuRD	↓	16-17
Epigenome		
Epigenetic clock	↑	43
Other changes	↑	44
Telomere		
Telomere shortening	↑	45
Telomere elongation	↓	45
Mitochondria		
Mitochondrial biogenesis	↓	16-17, 46
Mitochondrial function	↓	16-17, 47-48
Bioenergetics	↓	49
ΔΨm	↓	50
ATP	↓	51
NAD^+^	↓	52-53
TCA cycle	↓	54
Glycolysis	↑	55-56
Deregulated Signaling		
mTOR	↑	57-58
NFκB	↑	59
Nutrient signaling	↑	16-17, 48
Omics		
Transcriptome	↓↑	60-61
Acetylome	↓↑	60
Proteome	↓↑	16-17, 60, 62
NAD^+^ metabolome	↓	54
αKetoglutarate metabolome	↓	54
Robustness of Housekeeping Function		
Ubiquitin Proteasome System (UPS)	↓	63
Autophagy	↓	64-66
Mitophagy	↓	66-67
Robustness of ER Stress Response		
Unfolded protein response (UPR)	↓	68-69
Cell fitness		
Robustness of circadian rhythms	↓	70
Proliferation, regeneration and increased population doublings	↓	71-73
Lost functions	↑	73
Cell signaling	↓↑	74-75
Damage Response		
Oxidative-ROS	↑	76-77
DNA	↑	67, 78-80
Cancer	↑	81-82
Repair Response		
Cell and tissue repair	↓	83
PARP activity	↑	84
Inflammatory Response		
Inflammatory signaling	↑	85-86
Senescence		
Hallmarks of senescence	↑	87-89
SASP	↑	90
Stem cells		
Exhaustion	↑	16-17, 48
Cell Death		
Apoptosis	↑	91

↑ Improved or protected, ↓ Deteriorated or reversed, Mutations or other changes that are unlikely to be reversible are not included.

Rejuvenins, might be considered as part of the innate defense mechanisms that have been evolutionary designed and evolved to prolong life as much as possible and to prevent catastrophic early failures of cells, tissues, and organs, as they occur in progeroid syndromes. In contradistinction, late loss of these molecules can significantly prolong life in octogenarians (80-89 years), nonagenarians (90-99 years), centenarians (100-109 years) and super-centenarians (110 years and older).

There are several conditions that lead to cell rejuvenation and resetting of biologic and epigenetic clocks (Fig. 1D). In all such cases, restoration of old cells to more youthful cells and tissue phenotypes have been associated with the molecular signatures of youthfulness.

### Rejuvenation by fertilization and parthenogenesis

The most pronounced form of rejuvenation occurs after each fertilization. The cytoplasm of a zygote holds factors that are capable of resetting the epigenetic clock. In mouse, within 6-8 hours after fertilization, paternal genome, actively undergoes extensive demethylation, whereas the demethylation of maternal genome is initiated after several cell divisions [141,142]. The reprogramming in DNA methylation continues in early stages of embryogenesis, leading to rejuvenation and allowing the organism to start life at an earliest feasible epigenetic time-point (Fig. 1D) [143]. The rejuvenation also occurs when the egg is artificially inseminated or even when the animal is cloned by introducing somatic cell nuclei into a surrogate egg (somatic cell nuclear transfer; SCNT) and then the hybrid cell receives electric shock (parthenogenesis) instead of sperm (fertilization) to initiate embryogenesis [144-146]. The renewal after each fertilization, therefore, shows, that the biologic age of a cell can be completely reset and that most, if not all evidence of damage to cellular compartments, that are detriment to a normal lifespan, can be erased.

**Table 2:** Impact of aging and senescence on clinical parameters

Item	Effect	Reference
Blood and Bone Marrow		
Acid base balance	↓	92
Blood Lipidome	↓↑	93-94
Blood Metabolome	↓↑	95-96
Blood pressure	↑	97
Bone marrow function	↓	98-100
Integrity of endothelial barriers	↓	16-17
Bone		
Bone mass	↓	101
ECM		
ECM biosynthesis and organization	↓	102-103
ECM degradation	↑	104
Energy and Metabolism		
Glucose tolerance	↓	105-106
Energy utilization - Appetite and stamina	↓	107-110
Fat depots	↓	111
GI tract		
Integrity of GI barrier (leaky gut)	↑	16-17
Immune Response		
Immune response (immunosenescence)	↓	16-17, 112
Inflammation	↑	113-116
Liver		
Liver metabolism	↓	117-118
Musculoskeletal System		
Muscle mass and physical frailty	↓	119
Nervous System		
Memory	↑	120
Neural plasticity	↓	121
Integrity of BBB	↓	122
Cognition	↓	110, 122
Sleep	↓	124
Thermoregulation	↓	107, 125, 126
Reproductive System		
Fertility	↓	127, 128
Respiratory System		
Integrity of respiratory tract barrier	↓	16-17
Oxygenation	↓	107, 130-131
Skin		
Integrity of Skin Barrier	↓	16-17
Stem Cells and Tissue Regeneration		
Stem cell function	↓	16-17, 132-134
Tissue regeneration	↓	132, 135, 136
Organ Protection		
Organ, tissue, and metabolic protection	↓	16-17, 110
Senescence		
Senescence	↑	115, 137
Aging		
DNAm Age	↑	44
Age-related disorders and disease	↑	88, 110
Cancer		
Risk of cancer	↑	81, 138
Health-span and Lifespan		
Health-span	↓	139
Lifespan	↓	139, 140

↑ Improved or protected, ↓ Deteriorated or reversed, Mutations or other changes that are unlikely to be reversible are not included.

It has been known that chronological and biological age do not necessarily match, and some individuals appear and function much better than others who are at the same age. The proof for this observation was achieved by the pioneering work of Horvath. Horvath *et al* examined the DNA methylation status of 353 genes in 8,000 samples from 51 healthy tissues and various cell types and showed that the epigenetic age can accurately estimate the age of any tissue within a narrow 2-year margin and can be used as a great predictor of heritable acceleration of age [44, 147, 148]. Multi-tissue clock of Horvath also showed that, in human embryonic stem cells (ESCs), the epigenetic age is near zero even after extensive passaging [44]. More recently, Kerepesi *et al* confirmed these findings and using rDNAm determined the epigenetic age of these cells to be mostly below zero [143]. These cells did not age even after 100 passages indicating that cells, equivalent to those in the early stages of embryogenesis, are essentially immune to aging. The epigenetic age in embryonic stem cells which has been consistently found to be below zero, starts ticking during mid-embryonic development in mouse and humans [143]. In mouse, for example, at E6.5 and E7.5, the epigenetic age was significantly lower than the age of cells at E13.5 (primordial germ cells, the direct progenitors of sperms and oocytes). Thus, the epigenetic age is initially diminished and then it increases from birth, and it consistently advances throughout life in virtually all tissues [143]. These findings show that it is possible to stop and reverse the aging processes in somatic cells if the conditions that exist in embryonic cells can be defined and applied to aging cells.

### Rejuvenation by genetic reprogramming

The epigenetic rejuvenation by fertilization can be simulated and the epigenetic age can be set to near zero by inducing genetic reprogramming (iPSCs) [41]. However, one of the side effects of therapeutically using iPSCs is that these cells form teratomas *in vivo* [149]. Moreover, C-Myc gene is an oncogene that reduces lifespan, and its decrease is associated with increased health-span [148]. Using such an insight, it was recently shown that introduction of Oct4, Sox2, and Klf4 genes (OSK) without cMyc to old mice was sufficient to erase signs of aging [150]. This reprogramming induced in the recipient old mice, a more youthful gene expression pattern. Moreover, after optic nerve crush injury, the treatment promoted axon regeneration and restored vision in a mouse model of glaucoma [151]. Finally, there was evidence of resetting of the DNA methylation age of retinal ganglion cells. The reprogramming was DNA demethylase (Tet1 and Tet2) dependent suggesting that DNA methylation patterns might not be merely co-incidental effects rather they play a causative role in aging. It is also becoming apparent that aberrations of DNA methylation are initiated by double stranded DNA breaks (DSB) that cause persistent age dependent epigenetic drifts in yeast to mammals [152]. Together, these studies have demonstrated that, the genetic reprogramming simultaneously erases differentiation [de-differentiation), causes telomeric rejuvenation and leads to resetting of the epigenetic clock to an earlier time-point. Thus, it appears that, similar to differentiation, aging is plastic and subject to reversal to a more youthful state [153-156]. Interestingly, a backup record of youthful epigenetic information, encoded in part by DNA methylation, appears to exist in mammalian tissues, that is used for restoration of a youthful epigenome [151, 155]. A clear question that arises is whether the de-differentiation can completely be separated from the epigenetic clock reprogramming.

Due to the fear of inducing tumors, Ocampo *et al*, introduced Yamanaka OSKM factors [Oct4, Sox2, Klf4, and c-Myc) to mouse and human cells under conditions that allowed their cyclic expression [156]. This partial reprogramming was shown to lead to rejuvenation, reversed the cellular and physiologic hallmarks of aging and prolonged the lifespan of a progeroid mouse model that undergoes premature aging [156]. This landmark study showed that, not only individual cells, but the entire organism can be successfully rejuvenated, giving credibility to the notion that rejuvenation of organisms is quite feasible and is within reach. Moreover, this type of reprogramming was not associated with an increased risk of tumorigenesis. Besides genetic reprogramming, the conditioned media or the so-called secretome of stem cells of diverse origins appear to include factors that can rejuvenate tissues. For example, the conditioned media of human umbilical cord or bone marrow derived mesenchymal stem cells rejuvenated human skin [157-158]. Such findings clearly indicate that rejuvenating factors are released and exist within the stem cell secretome. Identifying such factors, therefore, offers the opportunity for rejuvenation of aging human cells.

The studies on epigenetic clock have made it clear that the trajectory of the epigenetic clock differs in different individuals and can be accelerated in certain conditions such as progeria. If the same variation would exist in different tissues of the same individual, the divergence of epigenetic age in different tissues would have ultimately emerged with time [41]. However, aging occurs uniformly throughout the body as evident by the similarity of aging of skin in the left and right sides of the body. The definitive proof for such a uniformity, was provided only recently by examining the epigenetic age of 30 anatomic sites in young people to supercentenarians. The findings confirmed that only the cerebellum and, to a lesser extent, the occipital cortical regions, exhibit negative epigenetic age acceleration in the supercentenarians [157]. DNA methylation (DNAm) age, in a host of sorted cell types (CD4^+^ T cells, monocytes, B cells, glial cells, and neurons, osteoblasts and osteocytes) as well as diverse organs (brain, breast, kidney, liver, lung, bone), and even the body fluids (whole blood, and saliva) that harbor DNA showed that the “epigenetic clock, uniformly applies to all of these cells, organs and body fluids [159]. Thus, there is a synchronicity in the epigenetic clock throughout the entire body and in the brain. Despite this knowledge, it has remained unclear as what accounts for the precision of the clock, or why its speed varies in different individuals or why and how the uniformity of age in all organs and tissues occurs. One possible explanation is that there are factors that circulate the blood to ensure that the epigenetic clock tick rate is the same throughout the entire body. Moreover, given that brain also ages coordinately with the rest of the body, we argue that the rejuvenating factors must also pass the BBB.

**Figure 2.**
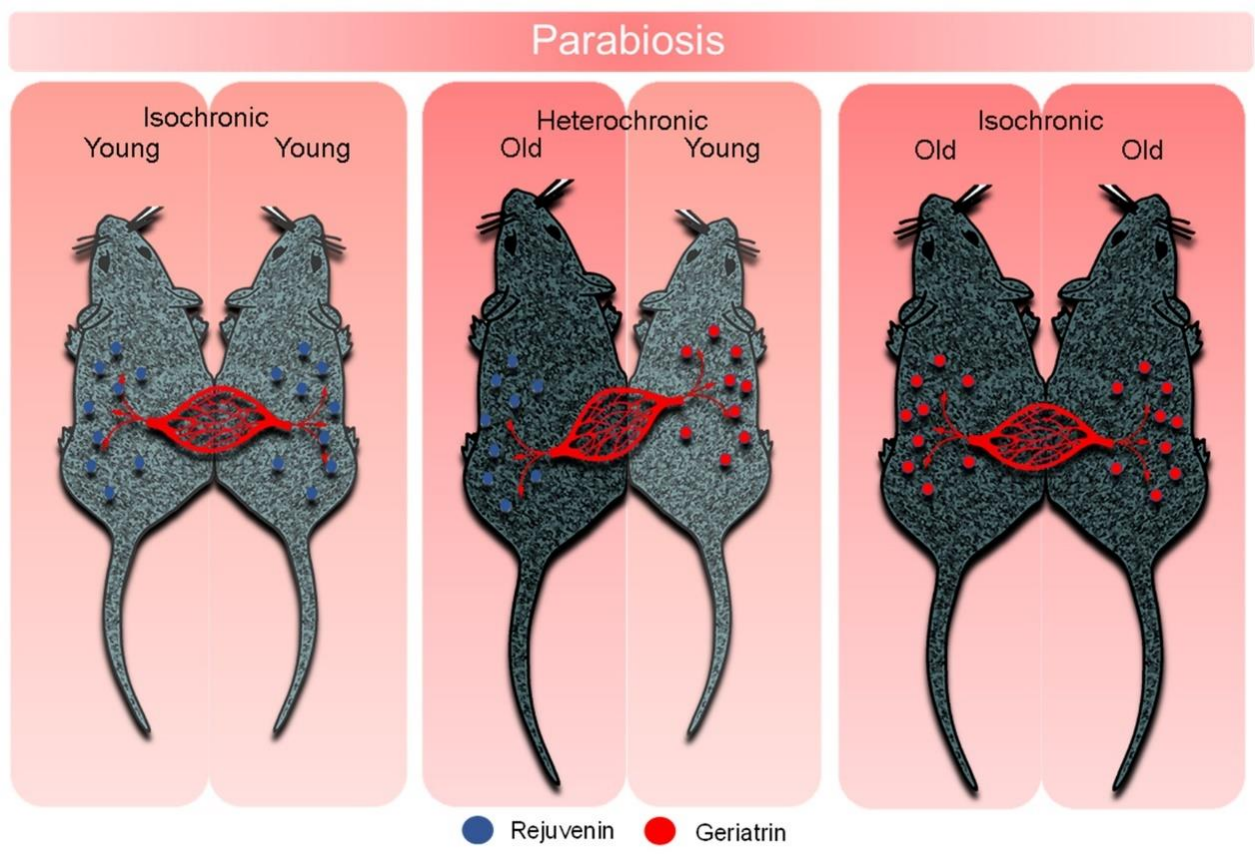
Isochronic and heterochronic parabiosis in mice. In young-old pairings, the blood of young mice has putative rejuvenating factors (rejuvenins) that lead to the rejuvenation of old mice whereas the blood of old mice has putative age inducing factors (geriatrins) that induce age-related disorders.

### Rejuvenation by parabiosis

The idea that circulating rejuvenating factors exist in blood and CSF is supported by studies that join two animals by their vasculature (parabiosis). Parabiosis is a technique that surgically joins the circulatory systems of two living organisms such as rats and mice so that they will have a single shared circulatory system [160-162]. In rats, the total blood that is exchanged between the two co-joined animals, reaches 10 times of the total volume of blood per day. This ensures that the two animals continuously receive the same level of blood constituents [163]. The pair of joined animals may be of the same age (isochronic parabionts) or can differ in age (heterochronic parabionts) (Fig. 2). Parabiosis defined as “living besides or next to” is one of the oldest surgical techniques which was first carried out close to two centuries ago by Paul Bert by surgically joining two rats, to simulate a condition, similar to the shared circulation of “Siamese twins” [164, 165]. Parabiosis has been used for over 150 years to test whether systemic or circulatory factors from one animal can affect another animal. For example, the discovery of leptin is owed to parabiosis experiments designed to identify whether a “satiety factor” circulates the blood [166]. Parabiosis was neglected until it gained popularity nearly hundred years later. McKay *et al* showed that in the heterochronic parabiosis, old parabionts gain life-extension, a finding that was validated by Ludwig *et al* in 1972 who showed a 20% increase in lifespan in old parabionts [167-168]. In these studies, young parabionts suffered negatively from the joined circulatory system perhaps because old blood might dilute the concentration of rejuventating factors present in young blood. However, a more likely possibility is that there are gero-inducing factors (geriatrins) that are produced by aging that exert negative effects on cell health causing the rampant aging deficits. Consistent with the latter possibility, the administration of plasma from old mice inhibited hippocampal neurogenesis in young mice *in vivo* and inhibited the function and proliferation of neural stem cells (NSC) *in vitro* [162]. These findings underscore the existence of molecules in the blood of old organisms that oppose or reverse the effect of rejuvenins and induce an aging phenotype (geriatrins).

The idea to join the circulatory systems of a young and an old animal made the parabiosis an impressive tool to examine the aging and age-related diseases in such contexts as diabetes, cardiovascular disease, Alzheimer’s disease, multiple sclerosis, and osteoarthritis [161, 162,169, 170]. These studies have repeatedly led to the conclusion that co-joining of young and old mice causes the rejuvenation of old animals and aging of the young organisms. These studies give credence to the existence of humoral factors in the circulatory system of young organisms that have rejuvenating (rejuvenins) or age inducing (geriatrin) effects [169, 171].

The effects of heterochronic parabiosis on aging involves every organ and tissue in the aged animal. The wide range of rejuvenating activity in heterochronic parabiosis is evident in rejuvenation of aged progenitor and stem cells and skeletal muscle stem (satellite) cells, to an increased density of the dendritic spine of mature neurons and synaptic plasticity in the hippocampus [161, 162, 172]. Heterochronic parabiosis of young and old mouse pairings led to restoration of the cEBP-alpha complex in old parabionts to levels that are seen in young animals and resulted in an increase in proliferation of hepatocytes in aged mice [161]. In a similar model, when compared with satellite cells from aged-isochronic controls, satellite cells in aged heterochronic parabionts exhibited improved myogenic differentiation and lower levels of DNA damage [173].

Using a limiting-dilution, competitive trans-plantation method, it was observed that the frequency of marrow competitive repopulation units (CRUs) increased approximately 2-fold in mice from 2 months to 2 years of age. However, the homing efficiency of old CRUs was approximately 3-fold lower than that of young CRUs. Moreover, the increased age of HSC donors and recipients promoted myelopoiesis [174-177]. However, heterochronic parabiosis led to the restoration of bone marrow function and dramatically reduced long-term HSCs to their normal ‘youthful’ levels.

The notion that young environments have a rejuvenating potential was demonstrated by restoring the regenerative potential of old muscles when transplanted to young rats. However, the young muscles showed declined regeneration when they were grafted to old rats [178]. In both isochronic and heterochronic parabioses, only five days after induction of muscle injury, muscles in young animals regenerated. Whereas injured muscles from old isochronic parabionts failed to regenerate, the heterochronic parabiosis of old and young mice restored the regenerative potential of aged muscles, an effect that was attributed to notch ligand that was shown to be required for muscle regeneration [179].

### Rejuvenation by reactivation of telomerase

One of the key features of aging is progressive attrition of telomeres, mainly due to the loss of telomerase activity and their uncapping, which then leads to stem cell depletion and consequently, to progressive tissue atrophy, organ system failure and impaired response to tissue injury [183-181]. In aging mice that harbored short telomeres, increased DNA damage signalling and degenerative phenotypes, telomerase reactivation, reduced the DNA damage signalling and cellular checkpoint responses and restored lost proliferative potential in quiescent cells. This reactivation also eliminated degenerative phenotypes in multiple organs including testes, spleens, and intestines. Moreover, the treatment also led to the reversal of age-related neurodegeneration as evidenced by the restoration of proliferating Sox2^+^ neural progenitors, and Olig2^+^ oligodendrocyte populations [181]. Unfortunately, the reactivation of telomerase is not clinically suitable for human use since telomerase activation following telomere dysfunction can lead to carcinogenesis [182].

### Rejuvenation by modifying cell signaling

Molecular approaches have allowed pinpointing the cell signaling pathways that have gone awry in aging. Reversal of these aberrant signalings is sufficient to restore homeostasis and reverse age-related declines. Most notably among these, is the activation of NFκB that drives inflammatory response and of mTOR which senses the cellular nutrient levels and regulates the rate of protein synthesis and energy utilization.

Conditional inhibition of NFκB in the epidermis of chronologically aged mice for 2 weeks, restored the gene expression programs that are found in young mice and erased the molecular signature of aging including SA-β-gal activity, *p16* down-regulation, and p16INK4A protein expression [183]. This blockade also promoted the proliferation of skin progenitors and their normal differentiation and induced a more youthful skin phenotype that was associated with an increased skin thickness [183].

Similarly, inhibition of mTOR in old mice with rapamycin prevented the age-related increase in hematopoeitic stem cells or their expression of aging biomarkers, reversed aging-related declines in the function of mouse HSCs and enhanced the immune response to infection with influenza virus [184-185].

### Rejuvenation by chemical, diet, and life-style changes

Aging reversal has also been tested with a cocktail of specific factors including recombinant human growth hormone (hGH), combined with metformin and DHEA. The participants, initially, received for a week, hGH alone (0.015 mg/kg/day) and then 50 mg/day DHEA in the second week and finally, these were administered jointly with 500 mg/day metformin in the third week. By the fourth week, all doses were individualized based on particular response of each participant [186]. This treatment led to the improved immunological response and risk indices and reversed the epigenetic clock. The rate of reversal of the epigenetic aging relative to the actual chronological age increased from -1.6 year/year during the 0-9^th^ months to -6.5 year/year in the 9-12^th^ months of the study. This is the first report that the epigenetic age estimator of human lifespan can be reversed by an anti-aging strategy by drugs and hormones. Epigenetic age also responded to diet and life-style changes and dropped by an average of 1.96 years by the end of an 8-week treatment [187].

### Rejuvenation by extracellular matrix (ECM)

ECM does not merely act as a scaffold, it actively participates in the regulation of cellular fate, behavior, and function. Moreover, to maintain tissue functionality, the tissue microenvironment may have rejuvenating or age inducing effects in the cellular repertoire of that tissue [161]. For example, ECMs that were deposited by the young fibroblasts derived from either newborn or a 6-year-old boy, restored the functions that were lost in senescent fibroblasts [188]. Exposure of senescent fibroblasts to young ECM reduced the level of the intracellular ROS levels and senescent markers such as β-gal, and inhibitors of proliferation namely, p21Waf1, p16INK4a, p53 and caveolin-1. The treatment also led to the telomere extension, reduced the level of DNA breaks, increased mitochondrial membrane potential (ΔΨm) and restored a youthful morphology. Restored cells regained the telomere related DNA repair protein dimer, Ku, in a SIRT1 dependent manner, and started to proliferate within 7 days. In contrast to senescent cells that fail to respond to stimulation by epidermal growth factor (EGF), the rejuvenated cells regained their EGF responsiveness and exhibited phosphorylation of signal-regulated kinase (ERK) in response to EGF [188].

Together, these findings support the idea that there are rejuvenating factors [rejuvenins) in young organsisms that are lost upon aging and there are humoral factors that lead to a diseased landscape. The properties that can be gleaned from the available data and ascribed to rejuvenins and geriatrins include effects on various cell and clinical parameters that determine a healthy or aging status (Tables 1-2).

### Rejuvenins and geriatrins

We have adopted the terms, rejuvenins and geriatrins, for small endogenously produced factors, that circulate blood and are released from bone marrow, stem cell niches, ECM, muscle, nervous system, or other sites. Rejuvenins have rejuvenating and geriatrins exhibit age inducing effects. We use increase in health-span and lifespan as primary and other features of health and aging as secondary criteria to choose a host of rejuvenins and geriatrins discussed here. We discuss carnosine, polyamines, NAD^+^ and its precursors, melatonin, and alpha ketoglutarate (AKG) as rejuvenins and review the homocysteine (Hcys) and reactive oxygen species (ROS) as geriatrins.

Support for existence of rejuvenating factors that circulate blood is recently provided by the administration of young plasma to old rats that more than halved the epigenetic age of their blood, heart, and liver tissue and reduced senescence in various tissues [189]. Such studies confirmed that rejuvenins can reset the peripheral epigenetic clocks. Existence of circulating factors that can pass the *B*lood-*B*rain *B*arrier (BBB) was shown by heterochronic parabiosis experiments since a statistically significant rejuvenation also occurs in the brain of heterochronic parabionts, manifested by increased density of the dendritic spine of mature neurons and synaptic plasticity in the hippocampus [161, 162, 172]. In similar pairings, the old parabionts showed an increase in volume and blood flow in cerebral blood vessels, a higher self-renewal and differentiation of neural stem cell population in subventricular zone and exhibited improvement in olfactory discrimination [190]. Consistent with these findings, administration of plasma from young mice into aged mice reversed cognitive decline, including contextual fear conditioning and spatial learning and memory [162]. Thus, entry of rejuvenins to the brain ensures that the epigenetic clocks in the brain and the rest of the body are in tune and synchronized in terms of their tick rate. The idea that rejuvenins or geriatrins must pass BBB, restricts the type of molecules that have age altering property to small soluble factors. The size of molecules that pass the BBB, is estimated to be as small as 100-400 dalton (Da) to a maximum of 1000 Da. Small molecules generally cross the BBB in pharmacologically significant amounts only if the molecular mass of the drug is less than 400-500 Da, and the compound forms less than 8-10 hydrogen bonds with solvent water [192]. To modify the cell function, they must also be sufficiently small that either can pass the plasma membrane freely or enter the cell by transporters that exist within cell membrane [193]. In fact, the molecular weights of our chosen candidates all fall below the maximum of 1000 Da (Table 3). If any of these factors is a metabolite, then, the predicted molecular weight is estimated to be less than 1500 Da [194-196]. However, large polypeptides and proteins due to their size, charge and reduced mobility are unlikely to act as a rejuvenin or geriatrin.

**Table 3:** Molecular mass and chemical composition of rejuvenating factors.

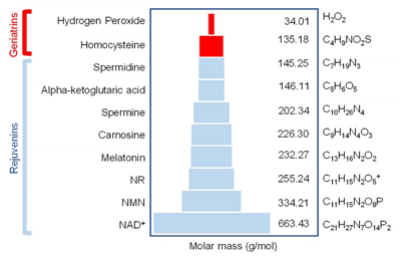

Rejuvenation is short-lived and continuous release of rejuvenating factors is required to maintain a youthful phenotype and their gradual loss can progressively result in the development of an aging phenotype. In other words, rejuvenation is plastic, requiring active maintenance, and withdrawal of rejuvenins promotes aging. Thus, aging might be equivalent to atrophy which occurs when the trophic factors are withdrawn with the exception that geriatrins further exacerbate the impact of rejuvenin withdrawal (Fig. 1B). OKSM factors also are required to be continuously administered since only days after their withdrawal, their rejuvenating activity ceases [156]. In a rejuvenation trial in humans that were treated with rGH, metformin and DHEA, consistent with the transient nature of rejuvenation requiring active maintenance, when the treatment was discontinued, the GrimAge predictor of human morbidity and mortality persisted only for six more months [186]. Similarly, the aging phenotype also appears to require constant maintenance. For example, continuous NF-κB activity is sufficient to induce, in the mouse skin, a gene expression pattern that is reminiscent of those induced by aging [183]. More importantly, blocking NF-κB activity reverses many of the characteristics of aging in skin of old animals. Thus, continuous activity of NF-κB is required to actively maintain the aging phenotype and withdrawal of such a blockade is sufficient to erase the aging phenotype rendered by continuous NF-κB activity [183]. This suggests that continuous presence of geriatrins that induce chronic NF-κB activation and nuclear translocation would be required for sustaining the inflammaging. The fragile nature of youth and aging requiring active maintenance is also evident in experiments that used young ECM. The proliferation of rejuvenated old cells that were deposited on young ECM was arrested after approximately 25 population doublings, and these cells developed senescence as evident by flattened and enlarged cell shapes and high SA-β-gal activity showing that youth must be maintained, and that loss of youthful factors permits a down-hill fall into the abyss of an aging phenotype [188]. These findings support the idea that the significant fall in serum levels of all rejuvenins with aging and coordinate increase in geriatrins is likely permissive for aging to be settled (Fig. 1).

Rejuvenins are expected to show a broad range of rejuvenating properties that reverse those functions that deteriorate with age. The age induced decline includes loss of cell and organellar fitness and cell functions, homeostasis, frailty, loss of cognitive functions, increase in blood pressure, disturbed glucose tolerance, altered barrier integrity, eroded functional omics, decreased proliferation responsive to local needs, attrition of telomeric length, decreased bioenergetics, oxidative and ER stress or DNA damage responses, reduced or loss of repair mechanisms, inflammatory signaling, senescence and increase in epigenetic age (Fig. 1, Tables 1-2).

Both circadian clock and methyl cycle evolved early in evolution in species as diverse as unicellular algae to humans [197]. Circadian clocks and methyl cycles are tightly linked. For example, circadian rhythms modify the global DNA methylation daily whereas inhibition of methyl cycle disrupts these rhythms [197]. Aging leads to progressive erosion of robustness of circadian rhythms and by progressive accumulation of cytosine modifications in DNA, aging likely modifies global and site-specific DNA methylations (Fig. 1) [115, 116]. This age induced loss of maintenance of the DNA methylation might be caused by the activity of TET in open chromatin, enhancers and CpGs which show dynamic circadian oscillations [148]. Thus, the rejuvenating effect of rejuvenins might, at least, partially, be due to their impact on regulation of circadian rhythms, epigenetic modifications, or both. For example, the secretion of melatonin is rhythmic, and its nocturnal release allows it to have rhythmic effect on circadian rhythms, sleep, and body temperature. Moreover, melatonin exerts effects on DNA methylations that are opposite of those which are inducible by light [198]. It appears that melatonin exerts its effect on methylation and regulates epigenome by inhibition of DNA methyltransferase [199]. Similarly, the levels of enzymes that participate in generation of polyamines and their levels undergo circadian oscillations. Polyamines, in turn, adjust the circadian period and their loss leads to longer periods [200]. Thus, rejuvenins are expected to regulate and oppose the changes in methylome, and circadian rhythms that occur with age, and should potentially be able to reset the epigenetic age and extend health-span and lifespan.

**Table 4:** Restoration of cell responses by treatment with Carnosine, NAD^+^, polyamines, and melatonin.

Cell Response	Carnosine	NAD^+^	Polyamines	Melatonin
Genome and epigenome				
DNA methylation	↓		↓	↓
Histone acetylation	↓		↓	
Telomere				
Telomere shortening		↓	↓	
Mitochondria				
Mitochondrial biogenesis		↑	↑	↑
Mitochondrial function	↑	↑	↑	↑
Bioenergetics		↑		↑
ΔΨm	↑		↑	
ATP	↓^*^	↑	↑	
TCA cycle	↑	↑		
Omics				
Transcriptome	↑			
Acetylome			↑	
Proteome			↑	
Metabolome		↑		
Housekeeping Function				
Ubiquitin Proteasome System		↑	↑	↓^*^
Autophagy/Mitophagy	↑	↑	↑	↑↓
ER				
Stress	↓	↓	↓	
Cell fitness				
Circadian rhythms	↑	↑	↑	↑
Proliferation	↑	↑	↑	↑
Population doublings	↑			
Restoration of lost functions		↑	↑	
Cell Signaling	↑	↑	↑	↑
Damage				
ROS stress	↓	↓	↓	↓
DNA damage	↓	↓	↓	↓
Oxidative damage	↓	↓	↓	↓
Senescence	↓	↓	↓	↓
Sirtuin activation		↑		
Cell damage and death	↓		↓	↓
Repair				
DNA repair		↑		↑
PARP activity	↑	↑		
Repair and regeneration	↑		↑	↑
Inflammatory Response				
Inflammatory signaling	↓	↓	↓	↓
NFκB activation and nuclear translocation				↓
SASP		↑^*^		↓

↑ Improved or protected ↓ Deteriorated or reversed ^*^ Not consistent with rejuvenation

Although the rejuvenin and geriatrin candidates that we discuss here, fulfill many of expected requirements, they do not exhibit identical properties, because data on some of their effects are still required (Tables 4-8). Below, we briefly discuss the salient rejuvenating features of some rejuvenins (carnosine, polyamines, NAD^+^, melatonin, AKG) and although there are other examples of geriatrins such as iron, for the purpose of brevity, we will discuss only two geriatrins (homocysteine, reactive oxgen species) [330-331]. Dyshomeostasis of iron in aging is associated with neuroinflammation, abnormal protein aggregation, neurodegeneration, and neuro-behavioral deficits [330].

### Carnosine

**Figure 3.**
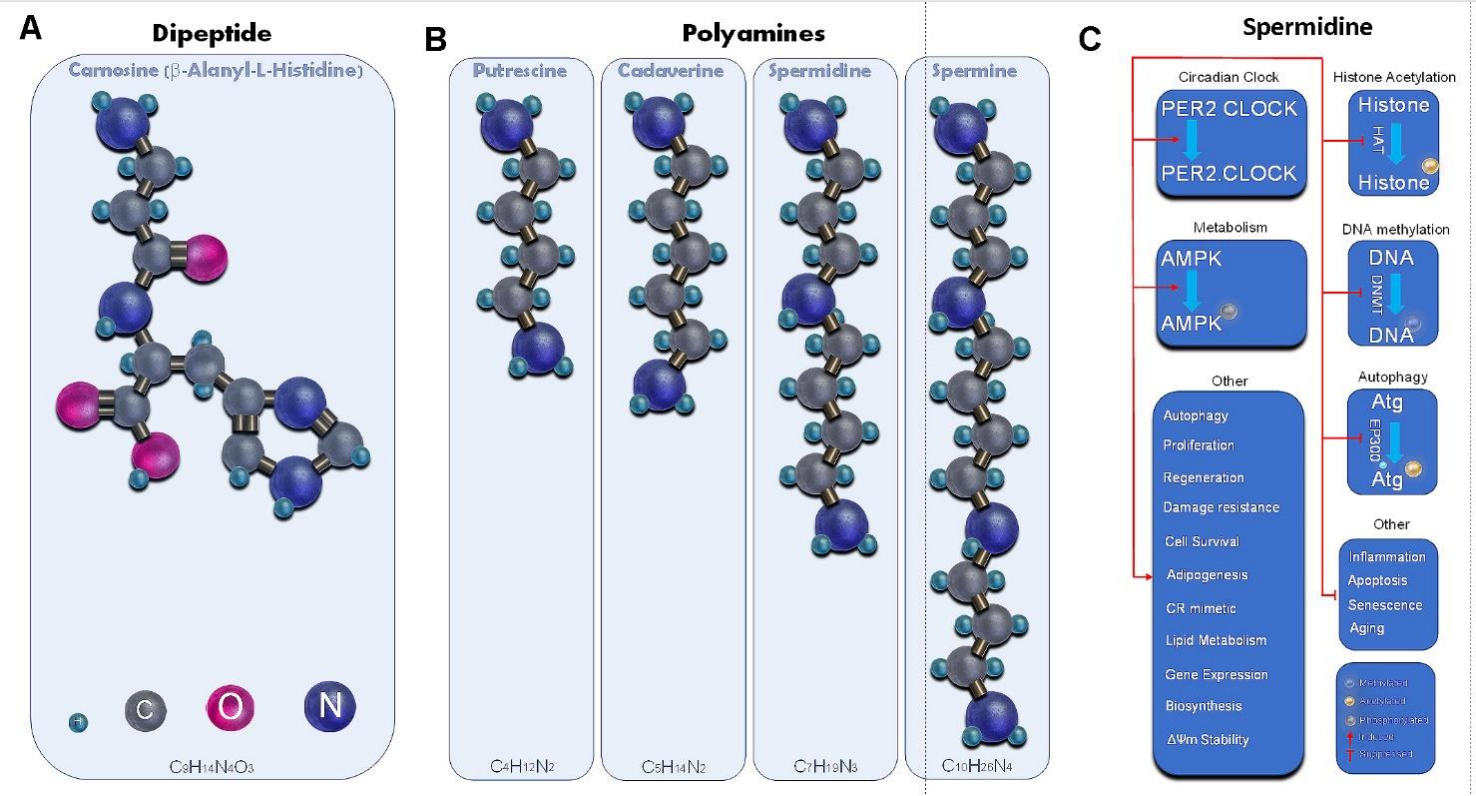
Structure of carnosin and polyamins and effects of spermidine. (A) Molecular structure of carnosine. (B) Molecular structure of polyamines. (C) Cellular and clinical effects of spermidine.

Carnosine is a small di-peptide (AH or β-alanyl-l-histidine) that was first discovered in 1900 by V.S. Gulewitch [332] (Fig. 3A). Carnosine is synthesized from β-alanine and L-histidine, by carnosine synthase (ATPGD1) which is present mainly in the heart and skeletal muscle, olfactory bulb and epithelium and olfactory receptor neurons and oligodendrocytes [333-335]. Carnosine is widely distributed in the body and is present in excitable tissues, most notably, the heart, the kidney, spleen, nervous system, olfactory bulb, choroid plexus, cerebral cortex, plasma, and CSF [332, 336]. Skeletal muscle is a depot and most prominent site for production of carnosine that releases it during exercise into the tissue interstitium [337].

Following synthesis, carnosine undergoes modifications including methylation, acetylation and decarboyxlation. A methylated variant of carnosine, either anserine (β-alanyl-Npi-methyl-histidine) or ophidine/balenine (β-alanyl-Ntau-methyl-histidine) are found in most animals but not in humans [332]. The production of methyl-anserine involves transfer of a methyl group from S-Adenosyl-Methionine (SAM) by N-methyltransferase (CMT) or by direct condensation of β-alanine with methyl-L-histidine [333-334]. The ophidine is produced by the enzymatic condensation of β-alanine with Npi-methyl-histine (or Ntau-methyl-histidine) by carnosine synthase. Acetylated form of carnosine (acetyl-carnosine) is produced by substitution of L-histidine with histamine. There are also different variants of carnosine that include homo-carnosine, and carnicine which is produced by the decarboxylation of carnosine [332].

Carnosine and its methylated analogs are transported through the cellular membrane by the proton-coupled oligo-peptide transporters (POT-family or SLC15). The mammalian family members include PEPT1 and PEPT2 (oligo-peptide transporter 1 and 2) and PHT1 and PHT2 (peptide-histidine transporter 1 and 2) [338]. Besides carnosine, the PEPT1 and PEPT2, transport 400 di-peptides and 8,000 tripeptides, however, these members do not transport amino-acids or longer peptides. In contrast, the PHTs, in addition to di-peptides and trans-peptides can transfer L-histidine [339-340]. Carnosine is ultimately degraded by serum carnosinase (CN1) and by tissue carnosinase (CN2) or by a non-specific cytosolic di-peptidase [341-343].

Carnosine is a major player of health and is essential to cellular homeostasis by protection from modifications of a large array of cellular functions that erode with aging (Tables 4-5). Carnosine and anserine are anti-fatigue agents, which, in honor of the discovery of the effect by Severin, is named the “Severin's phenomenon”. Carnosine protects muscles from fatigue during rhythmic nerve-stimulated muscle contractions by acting as a pH buffer. By such an action, carnosine limits the extent of blood acidosis that results from excess lactic acid which is produced by glycolysis during persistent muscle contractions (Boldyrev, 2013).

**Table 5:** Restoration of clinical responses by treatment with Carnosine, NAD^+^, polyamines, and melatonin.

Clinical Response	Carnosine	NAD^+^	Polyamines	Melatonin
Blood				
•Blood and serum profile	↑	↑		↑
•Blood pressure	↓			↓
•Bone marrow function	↑	↑		
ECM				
•ECM biosynthesis	↑	↑		
•ECM degradation	↑	↑		↑
Energy and Metabolism				
•Glucose tolerance	↑	↑	↑	↓^*^
•Energy	↑	↑		↑
Immune Response				
•Immune response	↑	↑		↑
•Inflammatory signaling	↑	↑	↑	↑
Liver				
•Liver metabolism	↑	↑	↑	↑
Musculo-skeletal System				
•Bone mass	↑	↑		↑
•Muscle mass	↑	↑		↑
Nervous System				
•Cognition	↑	↑	↑	
•Memory	↑	↑	↑	↑
•Neural plasticity	↑	↑		
•Neuroprotection	↑	↑	↑	↑
•Sleep	↑	↑	↑	↑
Reproductive System				
•Fertility	↑	↑	↑	↑
Stem Cells and Tissue Regeneration				
•Stem cell function	↑	↑	↑	↑
•Tissue regeneration	↑	↑	↑	↑
Organ Protection
•Organ protection	↑	↑	↑	↑
Senescence				
•Senescence	↓	↓	↓	↓
Age-related Disorders				
•Age-related disorders	↓	↓	↓	↓
Disease				
•Risk of disease	↑	↑	↑	↑
Cancer				
•Risk of cancer	↓	↓		↓

↑ Improved or protected ↓ Deteriorated or reversed ^*^ Not consistent with rejuvenation

**Figure 4.**
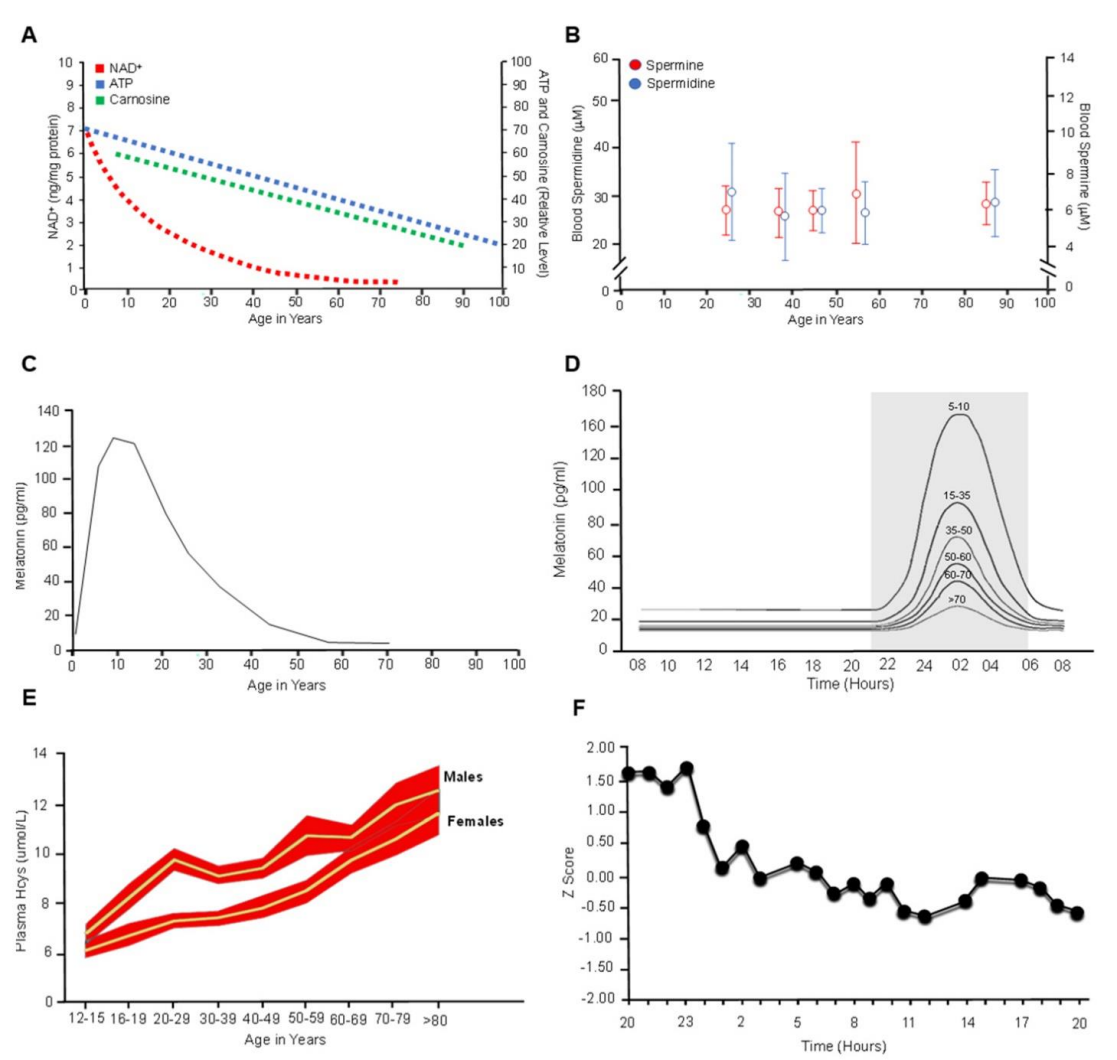
Day and age-related changes in the levels of rejuvenins. (A) Serum levels of NAD^+^, ATP and carnosine throughout life (349-353). (B) Serum levels of spermine and spermidine throughout life (404). (C) Serum levels of melatonin throughout life (499). (D) Serum levels of melatonin throughout a single circadian rhythm (354-355). (E) Plasma levels of Hcys throughout life in males and females (356). F. Plasma levels of Hcys during a 24 hr cycle (357).

Carnosine is protective of cell and tissue functions, effects that are gradually diminished as concentration of muscle carnosine and its serum levels decline with age or in age-related diseases such as Alzheimer’s disease (AD) [216, 217, 218, 220, 344-348] (Fig. 4A). However, such a drop can be compensated for by taking carnosine as a supplement since carnosine from food sources is absorbed by intestinal absorption by peptide transporter, PEP1 and its levels in blood is maintained by re-adsorbtion from renal tubules by PEP2 [332, 349-350]. In healthy volunteers, the daily ingestion of L-carnosine at 13 grams per day for 4 weeks elevated the muscle carnosine content by 65% or by 80-85% when taken orally for 10-12 weeks [332].

The wide range of biological activities of carnosine is attributable to its property in resetting the clock genes, (*Bmal1*, *Dec1*, *Cry1*). Carnosine also accelerates the resetting rate of the myocardial clocks by virtue of acting on the autonomic nervous system [351]. Carnosine influences the transcription of the pyruvate dehydrogenase kinase 4 (PDK4), an action that requires epigenetic regulation of PDK4 through enhanced histone acetylation of its promoter [352]. In eukaryotes, PDKs (PDK1-4) regulate the pyruvate dehydrogenase complex in the mitochondrial matrix that converts the pyruvate derived from glycolysis in the cytosol to acetyl-coenzyme A, that its oxidation by the Krebs cycle, produces energy in the mitochondria [361]. Carnosine leads to de-acetylations which are mediated by sirtuins [353].

Carnosine protects against ROS induced damage since it is an antioxidant, and defends the tissues against oxidative stress, peroxynitrite-dependent reaction, hypochlorite (HOCl) and ischemia-reperfusion damage in multiple organs such as brain, kidney and testis and regulates the blood pressure and systemic glucose levels [332]. Carnosine, at physiological concentrations, directly reacts with superoxide anions like superoxide dismutase (SOD) and quenches peroxyl radicals, O2^·-^, and ROS such as hydroxyl radicals (^·^OH) [354, 355].

The anti-aging mechanisms of carnosine are attributed to its suppression of protein oxidation, glycation, AGE formation, and cross-linking, reducing the effects of reactive carbonyls, modulating mitochondrial function and autophagy, inhibiting the cellular respiration regulatory complex, and by acting as natural antioxidant, anti-inflammatory, and anti-senescence agent [356-360] (Tables 3-4). Carnosine prevents the age induced glycation of proteins that alters protein structure and decreases their biological activity. Carnosine prevents glycation and enhances the thermal un-folding of glycated proteins as validated by a decreased T (0.5) and a lowered Gibbs free energy barrier and enthalpy of denaturation, consistent with the idea that carnosine promotes hydration during heat denaturation of glycated proteins [361].

Carnosine protects tissues against protein carbonylation and glyco-oxidation, preventing the formation of advanced lipo-oxidation end-products (ALEs) and advanced glyco-oxidation end-products (AGEs) which are involved in oxidative damage in age-related pathologies including those which occur in diabetes, atherosclerosis, and Alzheimer’ disease. Carnosine reacts and quenches *in vitro* and *in vivo*, α, β-unsaturated aldehydes, such as HNE, the most abundant class of ALE precursors [332]. Carnosine, provided as supplement, reduces protein carbonylation in the kidney, lowers the level of urinary AGEs, and diminishes the collagen cross linking as evident by an increase in collagen solubility [362]. By reversal of protein glycation and inhibition of formation of AGEs and ALEs, carnosine prevents and reduces diabetic complications such as diabetic neuropathy and abnormal sensory perception. Carnosine also contributes to the removal of damaged proteins by acceleration of protein turnover by a rapid step involving a localized thermal un-folding, and then a slower proteolytic event [361, 363].

One of the age-related pathologies is the development of cataract, that results from cross linking of the major structural lens protein, crystallin (predominantly α-crystallin) and formation of insoluble aggregates. Carnosine prevents the self-assembly of alpha-crystallin, into fibrillar structures, under mild denaturing conditions *in vitro*, dis-aggregates glycated α-crystallin, ensures the lens transparency, protects the lens from the development of cataract and delays the progression of cataract in diabetic rats [364-366]. Application of ophtalmic solution of N alpha-acetyl-carnosine (NAC), which is resistant to the enzymatic hydrolysis by human serum carnosinase, has shown improvement in visual acuity and glare sensitivity [367, 368].

Carnosine protects against diabetes by its anti-oxidative property, exerts glycemic control, reduces plasma glucose and hyperglycemia, by increasing the insulin levels and by preservation or increase in the number of β-cells in the pancreas [332]. The protecting effect of carnosine on myocardial fibers is due to increases in neutral (non-lysosomal) protease activity [369-370]. In cardiovascular system, carnosine enhances cardiac contractility and shows anti-hypertensive and hypotensive effect, likely by vasodilation, through carnosine-histidine-histamine pathway, or NO-cGMP mechanism, or directly by the modulation of the autonomic nervous system [332]. Carnosine protects kidneys from podocyte loss and from diabetic nephropathy and by reducing the expression of Bcl-2-associated X protein (bax) and cytochrome *c* protects glomeruli from apoptosis [371-373]. Carnosine also protects tissues from damage such as prevention of mucosal ulcerations or *Helicobacter pylori* induced gastritis and of age-related pathologies in various organs [332].

Carnosine has protective effects against AD as evidenced by inhibition of polymerization of β-amyloid, and the neurotoxicity of amyloid-β [374]. In 3xTg-AD mice, that develop a neuro-degenerative phenotype, that is similar to AD, carnosine reduced the hippocampal intra-neuronal accumulation of β-amyloid (Aβ) and fully restored mitochondrial dysfunctions [375]. Similarly, in transgenic AD mice, on a high fat diet, carnosine prevented cognitive decline [376]. In animal models of Parkinson’s disease, carnosine inhibited the oligomerization of α-synuclein and, by virtue of its anti-oxidative and anti-inflammatory protection of the striatum, prevented the development of Parkinson’s disease [377-379]. In a pilot study, it has been shown that carnosine (1.5 grams per day) increases the efficiency of DOPA therapy in patients with Parkinson's disease, decreases plasma protein carbonyls, increases SOD and improves clinical symptoms such as rigidity of hands and legs, leading to increased hand and leg movements [380].

Carnosine substantially improves the rate of wound healing by increasing cytokines and growth factors, collagen biosynthesis and fibroblast proliferation and increases the speed of healing of bleomycin-induced and irradiation-induced pulmonary wounds and in a model of type 2 diabetes [332].

**Table 6:** Associations of hyperhomocysteinemia with cellular and clinical features of aging.

Barrier Integrity		
•Impaired blood retinal barrier	↑	201
Cell Degeneration and Death		
•Apoptosis	↑	202
•Neural apoptosis	↑	203
•Apoptosis of mesenchymal stem cells	↑	204
Damage		
•Loss of function of cell membranes	↑	205
•Impaired genergy metabolism	↑	206
•Metabolome changes	↑	207
•Abnormal lipid metabolism	↑	208
•Alterations of serum VLDL	↑	209
•Defective DNA synthesis	↑	210
•Hypomethylation	↑	211
•Neural DNA damage	↑	212
•Inhibition of NO synthase (eNOS) expression	↑	213
•Inactivation of eNOS by over-expression of caveolin-1	↑	214
•Aberrant stem cell function	↑	215
Housekeeping Function		
•Decreased autophagy	↑	216
•Altered Ubiquitin proteasome composition	↑	217
Immune Response and Inflammation		
•Induction of inflammation and inflammatory markers	↑	218, 219
•Activation of NFκB	↑	220
•Release of inflammatory cytokines	↑	221
•Induction of monocyte chemoattractant protein-1 (MCP-1)	↑	220
•Induction of IL-8, chemokines, RAGE, MMP9 and adhesion molecules	↑	220, 222
•Monocyte proliferation	↑	221
•Monocyte activation	↑	221
•Immune activation	↑	222
Stress		
•Increase in ROS and oxidative stress	↑	223, 224
•Lipid peroxidation	↑	223
•Oxidative modification of low-density lipoproteins	↑	225
•Oxidative degradation of endothelial membrane lipids	↑	224
•Increased ER stress	↑	226
Induction of a thrombotic environment		
•Platelet activation	↑	227
•Induction of expression of tissue factor	↑	228
•Activation of factor V and VII	↑	222, 229
•Reduced protein C activation	↑	230
•Inactivation of expression of thrombomodulin	↑	231
•Formation of thrombus	↑	227
•Suppressing anticoagulant heparin sulfate expression	↑	232
•Blocking tissue plasminogen activator binding to endothelial cells	↑	233
•Depletion of endothelium-derived nitric oxide	↑	203
•Stimulation of N-methyl-D-aspartate (NMDA) receptors, resulting in calcium influx and excitotoxicity	↑	234
Bone		
•Decreased bone blood flow	↑	235
•Decreased formation of collagen cross links in bone	↑	236
•Decreased bone mass	↑	237
•Osteoporosis	↑	238
Barrier Integrity		
•Impaired blood retinal barrier	↑	201
Cell Degeneration and Death		
•Apoptosis	↑	202
•Neural apoptosis	↑	203
•Apoptosis of mesenchymal stem cells	↑	204
Damage		
•Loss of function of cell membranes	↑	205
•Impaired genergy metabolism	↑	206
•Metabolome changes	↑	207
•Abnormal lipid metabolism	↑	208
•Alterations of serum VLDL	↑	209
•Defective DNA synthesis	↑	210
•Hypomethylation	↑	211
•Neural DNA damage	↑	212
•Inhibition of NO synthase (eNOS) expression	↑	213
•Inactivation of eNOS by over-expression of caveolin-1	↑	214
•Aberrant stem cell function	↑	215
Housekeeping Function		
•Decreased autophagy	↑	216
•Altered Ubiquitin proteasome composition	↑	217
Immune Response and Inflammation		
•Induction of inflammation and inflammatory markers	↑	218, 219
•Activation of NFκB	↑	220
•Release of inflammatory cytokines	↑	221
•Induction of monocyte chemoattractant protein-1 (MCP-1)	↑	220
•Induction of IL-8, chemokines, RAGE, MMP9 and adhesion molecules	↑	220, 222
•Monocyte proliferation	↑	221
•Monocyte activation	↑	221
•Immune activation	↑	222
Stress		
•Increase in ROS and oxidative stress	↑	223, 224
•Lipid peroxidation	↑	223
•Oxidative modification of low-density lipoproteins	↑	225
•Oxidative degradation of endothelial membrane lipids	↑	224
•Increased ER stress	↑	226
Induction of a thrombotic environment		
•Platelet activation	↑	227
•Induction of expression of tissue factor	↑	228
•Activation of factor V and VII	↑	222, 229
•Reduced protein C activation	↑	230
•Inactivation of expression of thrombomodulin	↑	231
•Formation of thrombus	↑	227
•Suppressing anticoagulant heparin sulfate expression	↑	232
•Blocking tissue plasminogen activator binding to endothelial cells	↑	233
•Depletion of endothelium-derived nitric oxide	↑	203
•Stimulation of N-methyl-D-aspartate (NMDA) receptors, resulting in calcium influx and excitotoxicity	↑	234
Bone		
•Decreased bone blood flow	↑	235
•Decreased formation of collagen cross links in bone	↑	236
•Decreased bone mass	↑	237
•Osteoporosis	↑	238
•Risk of osteoporotic fracture	↑	239
Pancreas		
•Type 2 diabetes	↑	240
Muscle		
•Loss of muscle mass and function	↑	241
•Skeletal muscle atrophy	↑	242
•Inhibition of satellite regeneration	↑	243
Nervous System		
•Aberrant sleep	↑	244
•Neurotoxicity	↑	245
•Enhanced glutamine excitotoxicity	↑	246
•Enhanced β-amyloid neurotoxicity	↑	247
•Tau hyperphosphorylation	↑	248
•Cerebral cortical atrophy	↑	249
•Hipocampal atrophy	↑	249
•Disruption of frontal subcortical circuits	↑	250
•Disturbance in gait balance	↑	251
•Loss of motivation	↑	252
•Cognitive decline	↑	252
•Depression	↑	252
•Dementia	↑	252
Reproduction		
•Recurrent pregnancy loss	↑	253
Retina		
•Retinopathy	↑	254
Vessel Dysfunction and Disease		
•Hypertension	↑	255
•Angiotoxicity	↑	246
•Endothelial dysfunction	↑	222, 227
•Endothelial injury	↑	203
•Vascular smooth muscle cell proliferation	↑	256
•Depletion of endothelial dervied NO	↑	203
•Vascular disease (arteriosclerotic changes)	↑	257
•Cerebral micro-angiopathy	↑	258
•Cerberal macro-angiopathy	↑	258
•Proliferation of vascular smooth muscle cells	↑	256
•Coronary heart disease	↑	259
•Peripheral artery disease	↑	260
•Homocysteine-induced arteriosclerosis	↑	227
•Myocardial infarction	↑	261
Senescence		
•Induction of senescence	↑	262, 263
Risk of Disease and Cancer		
•Increased risk of degenerative disease	↑	264
•Increased risk for cancer	↑	265-266
Mortality		
•Increased risk of death	↑	267
•Increased risk of cardiac death	↑	268

↑ Increased or deteriorated

Carnosine prevents senescence, induces rejuvenation, and has geroprotective effect, likely by its wide spectrum of actions, including protein carbonylation and degradation of damaged proteins [381-383]. Carnosine also reduces the damage to telomeres and their shortening rate in fibroblasts [384]. Carnosine, at 50 mM, increases plating efficiency, the population doublings (PDs) and Hayflick limit, reduces senescence, and rejuvenates late passages of fibroblasts, a phenotype that is reversed upon carnosine withdrawal [356]. Carnosine mimics the geroprotecting action of rapamycin by inhibition of Akt/mTOR/p70S6K signaling [358, 385-386]. Carnosine extended lifespan in *Drosophila* and increased the lifespan by 20% in senescence-accelerated mice—prone 1 (SAMP1), leading to an increase in the number of animals that reached old age. These changes were associated with a decrease in the level of thiobarbituric acid reactive substances (TBARS), monoamine oxidase B (MAO B), and Na/K-ATPase activity [387-389].

Carnosine has been shown in mice to inhibit tumor growth and mortality, likely by inhibition of glycolysis which is essential to cancer growth (Warburg’s effect) by depletion of metabolic intermediates (glyceraldehydes phosphate and dihydroxyacetone phosphate), carbonyl quenching and reduced generation of ATP [390-393].

Together, the available data show that a small dipeptide has sweeping rejuvenating effects. These effects are found in cellular biology in terms of significant improvement in cellular fitness and damage control and repair. The clinical impacts of carnosine ensure maintenance of a youthful phenotype, by promoting longer health-span and lifespan expected from a true rejuvenin (Tables 4-5).

### Polyamines

Sperm and crystalline substances formed by semen were initially identified in 1678 by Antonie van Leeuwenhoek. These crystals were later shown to consist of a group of substances with multiple amine groups known as polyamines which include a diamine (putrescine; 1,4-diaminobutane), triamine (spermidine) and tetraamines (spermine and thermospermine) [394-395] (Fig. 3B). These natural polyamines are produced from ornithine by the action of ornithine decarboxylase (ODC) that converts it to putrescine which is then modified to spermidine by decarboxylation by S-adenosyl-L-methionine; (SPDSY). Spermine is produced by the action of spermidine synthase (SPMSY), and spermine synthase [396]. These polyamines are regulated by spermidine/spermine *N1*-acetyltransferase (SSAT) that causes their ubiqiutination and proteasomal degradation, an effect which can be blocked by the binding to polyamines [397].

Besides SSAT, other enzymes of the biosynthetic pathway of polyamines, all exhibit a rapid turnover ensuring that polyamines are produced in a time-limited fashion [397]. Moreover, the transcript levels of enzymes that participate in the biosynthesis of polyamines including ornithine decarboxylase 1 (*Odc1*), S-adenosylmethionine decarboxylase 1 (*Adm1*), and spermidine synthase (*Srm*), show circadian oscillations [398]. The hepatic circadian changes in the abundance of these enzymes as well as the circadian fluctuations of polyamines are lost in *Per1^-/-^Per2^-/-^* mice, that lack a functional circadian clock, showing that their circadian rhythms rely on *Per*. The level of the enzymes of the polyamine biosynthetic pathway starts to increase at ZT12, peaks at ZT16 and their levels drops significantly at ZT20 and ZT0 [399]. The daily oscillation of intracellular levels of polyamines has been confirmed *in vitro* in NIH/3T3 mouse fibroblasts and *in vivo* in the mouse liver [399]. In fact, coordinate with the levels of the enzymes participating in their synthesis (*Odc1*, *Amd1*, and *Srm*), the intracellular levels of putrescine and spermidine and not spermine increased and peaked at ZT16 [399]. The daily levels of enzymes that participate in polyamine synthesis are regulated through light driven rhythmic binding of *BMAL1:CLOCK* heterodimers to conserved DNA elements and in response to feeding. The polyamines, in turn, adjust the circadian period by regulating the interaction with and stabilizing *PER2*-*CRY1* heterodimers and for this reason, their loss during aging leads to longer circadian periods, a deficit that can be adjusted with polyamine supplementation [200, 399]. Also, spermidine regulates *BMAL1:CLOCK*-dependent transcription by virtue of inhibiting acetyltransferase E1A binding protein, p300 (EP300) [400]. By acting as a calorie restriction (CR) mimetic, spermidine also induces the beneficial effects of CR such as activating autophagy [401]. These data show an intimate relationship that exists between the daily circadian oscillations and the spermidine/spermine synthetic pathway and their polyamine output. Loss of this delicate balance artificially or by aging unleashes adverse impacts that can be abrogated only by supplementation of the diet with polyamines.

Due to their polycationic nature, polyamines have a wide range of effects since they readily interact with negatively charged molecules, including DNA, RNA, and lipids (Fig. 3C). There is evidence that polyamines regulate DNA methylation, and their effects have the potential to reverse the impact of aging on DNA. High polyamine intake that elevated polyamine concentrations in whole blood, prevented the development of age-associated genome-wide DNA methylations and coordinately reduced the age-associated pathological changes, inflammatory state, and mortality and extended lifespan in aged mice [402-404] (Fig. 4). Polyamines override aberrant DNA methylations and other changes that are caused by inhibiting ODC including increased decarboxylated levels of S-adenosylmethionine and decrease in DNA methyltransferase activity [404]. By virtue of its inhibitory effect on histone H3 acetylation by histone acetyl transferases (HATs), polyamines reduce ROS load and oxidative stress [405].

Polyamines participate in tissue regeneration by halting translation, increased cell proliferation and growth and by virtue of enhancing regenerative potential [406-408]. Inhibition of polyamine synthesis leads to disturbed cell cycle progression, within a single cell cycle, first affecting S phase and then G_1_ and G_2_/M phases [410]. In the yeast, *Schizosaccharomyces pombe*, spermidine, at a minimum concentration of 10^-6^ M was found to be an absolute requirement for growth and cell cycle progression [409]. Three of six spermidine homologs supported cell growth in presence of a specific inhibitor of putrescine biosynthesis, alpha-difluoromethylornithine (DFMO) [407]. As compared to α-methyl-ornithine, which is a competitive inhibitor of ornithine decarboxylase, its irreversible inhibitor, α-Difluoro-methyl-ornithine, which more potently inhibits polyamine production, coordinately inhibits growth [411]. L-α-Difluoro-methyl-ornithine that depletes the polyamines, reduces protein synthesis by inhibiting translation and thus, it seems that both translation initiation and elongation are dependent on presence of polyamines [412]. Due to their importance to tissue regeneration, coinciding with increase in DNA synthesis, the uptake of putrescine and the biosynthesis of spermidine, were accelerated in the liver within 2 hours after partial hepatectomy. Spermidine also promoted proliferation in stem cells of hair follicles, leading to hair shaft elongation and prolonged hair growth phase (anagen) showing that the effect of polyamines on cell growth and regeneration is not tissue dependent [408].

Autophagy represents cellular self-cannibalism, by which, non-nuclear cellular parts are transferred to lysosomes for degradation, and recycling, facilitating renewal of damaged molecules, organelles, and cytoplasmic and plasma membrane constituents. Aging leads to the loss of autophagy and genetic inhibition of autophagy has been shown to induce degenerative changes that are reminiscent of age-related pathologies [413-415]. Thus, it is clear that maintenance of such an important housekeeping function is required for tissue homeostasis and in preventing aging and age-related decline in autophagy as well as decrease in polyamine levels [416, 417] (Fig. 4B). Polyamines regulate autophagy by phosphorylating AMPK, which sustains ATP levels and by regulation of *MFN1*, *MFN2*, *DRP1*, and *COX IV* and by actions that involve both acetylation and SIRT1 independent deacetylation events by inhibition of EP300 [396, 400, 418]. Spermidine promotes autophagy by increasing ATG5 protein and LC3B-II levels, and by decreasing p62 protein expression [419]. The loss of cardiac autophagy leads to impaired heart function. Spermidine exerts cardio-vascular protective effects by improving the mechano-elastical properties of cardiomyocytes, by autophagy, mitophagy and by decreased vessel stiffness and reducing the mechanical load on the heart by lowering of the blood pressure [420, 421]. The cardio-protective effects of spermidine, at least in part, seem to be due to enhanced autophagy since such effects were not seen in ATG5 deficient mice [422]. In the brain, both spermidine and spermine induce autophagy in senescence accelerated mouse-8 (SAMP8) mice and delay brain aging as evidenced by increased activity of superoxide dismutase (SOD) that scavenges superoxide anions and by reducing malondialdehyde (MDA) in aging brains that is generated by ROS induced degradation of poly-unsaturated fatty acids [396]. Moreover, spermidine maintains brain health in older individuals who experience subjective cognitive decline [423].

Polyamines have anti-inflammatory and anti-apoptotic effects, e.g., spermidine supplementation was shown to reduce accumulation of neurotoxic soluble Aβ in early as well as late stages of Alzheimer’s disease [396, 424, 425]. In thioacetamide (TAA)-induced acute liver injury, spermine exerted its anti-inflammatory action by inhibiting M1 polarization, while promoting M2 polarization of Kupffer cells (KCs), decreasing their *STAT1* activation, reducing the levels of IL1-β, and iNOS, and increasing *STAT6* activation. The anti-inflammatory effect of spermidine in LPS activated macrophages is evident by reducing nuclear translocation of phorphorylated form of p65, and decreased production of ROS, NO, PGE_2_, TNF-α and IL1-β [426]. Similarly, in LPS treated microglial cells, spermidine blocked the nuclear translocation of p65, production of NO, and expression of TNF-α and IL-6 [427]. In skin, the anti-inflammatory impact of polyamines was shown by reducing edema, NO, TNF-α and IL1-β in 12-O-tetradecanoylphorbol-13-acetate (TPA)-induced dermal edema in mice [428]. In collagen-induced arthritis, spermidine was shown to reduce joint macrophage activation and inflammation in mice [429].

In the lungs, spermidine increased autophagy, reduced endoplasmic reticulum stress and cell death, and prevented bleomycin induced senescence as exemplified by decreased expression of *p16* and *p21*, β-gal, apoptosis, and fibrosis [430]. Spermidine afforded protection, retained locomotor activity, and increased survival in *Drosophila melanogaster* from toxic levels of superoxide generator, Paraquat, an action that required intact autophagy since these effects were absent in flies that lacked ATG7 [431]. Spermidine supplementation afforded more resistance to heat and hydrogen peroxide in yeasts and reduced oxidative stress in mouse fibroblasts in a glutathione independent manner [407, 432]. Spermine protected cells from DNA damage induced by Fenton reaction and at concentrations ranging from 0.1 to 1 mM, spermidine reduced release of 8-oxo-7,8-dihydroguanine and 5-hydroxycytosine after radiation and protected cells from radiation induced DNA damage [433, 434]. Spermidine, reduced retinal ganglion cell (RGC) death, prevented retinal degeneration and augmented optic nerve regeneration after optic nerve damage [435, 436]. Spermidine stabilized mitochondrial genome and membrane potential, reduced apoptosis and protected cells against D-galactose (gal)-induced mitochondrial damage [437].

Polyamines protect cells and organs against stress, prevent kidney from the damaging effect of diabetes, and reduce bone loss [438, 439]. In spermine oxidase mutant (Δspe3 Δfms1) strain of *Saccharomyces cerevisiae*, supplementation with spermidine or spermine led to the induction of transport-related genes and genes involved in methionine, arginine, lysine, NAD and biotin biosynthesis and down-regulated genes which are involved in nucleic acid metabolism and various stress responses [410]. Polyamines promoted wound healing through urokinase-type plasminogen activator (uPA)/uPA receptor (uPAR) signaling *in vitro* and in a skin wound repair *in vivo* [440].

Spermidine increases the lifespan of human peripheral blood mononuclear cells (PBMC) [405]. After 12 days in culture, only 15% of control PBMC survived, whereas up to 50% of the cells survived after treatment with 20 nM spermidine [405]. Similarly, supplementation of food with polyamines successfully increased the concentration of polyamines in blood, reduced the pro-inflammatory state that is typical of aged tissues [402]. This approach also prevented global alterations in DNA methylation, reduced age-related pathologies and decreased mortality in aged mice. The polyamine supplementation also increased lifespan and prevented liver fibrosis and development of hepatocellular carcinoma by activating MAP1S-mediated autophagy [402, 441]. In humans, high spermidine intake was associated with a lower rate of mortality and polyamine supplementation was shown to prolong health-span and lifespan in yeasts, flies, worms, to humans [402, 405, 431, 442, 443]. Polyamine depletion inhibited growth and caused cellular arrest in G1 phase, an event which was associated with increased p53 protein and other inhibitors including p21^Waf1/Cip1^ and p27^Kip1^. The treatment, within five hours, also led to up to 150% increase in the stress-activated protein kinase/c-Jun NH_2_-terminal kinase (JNK) type of mitogen-activated protein kinase (MAPK) and caused a sustained increase in the activity of ERK-2 isoform [444]. These findings suggest that the prolonged life in Nona-Centenarians, at least to some extent, might be owed to the high levels of polymaines in their blood [445].

The effects of spermidine on aging appears to depend on insuring DNA stability, fostering autophagy, promoting cellular fitness, cell growth and tissue regeneration, enhancing adipogenesis, reducing apoptosis, protecting against organ mal-function, and in enhancing anticancer immune surveillance [442]. The effectd of polyamines are ultimately translated to reducing age-related decline by promoting health-span and lifespan [431].

### NAD^+^ and its precursors

**Figure 5.**
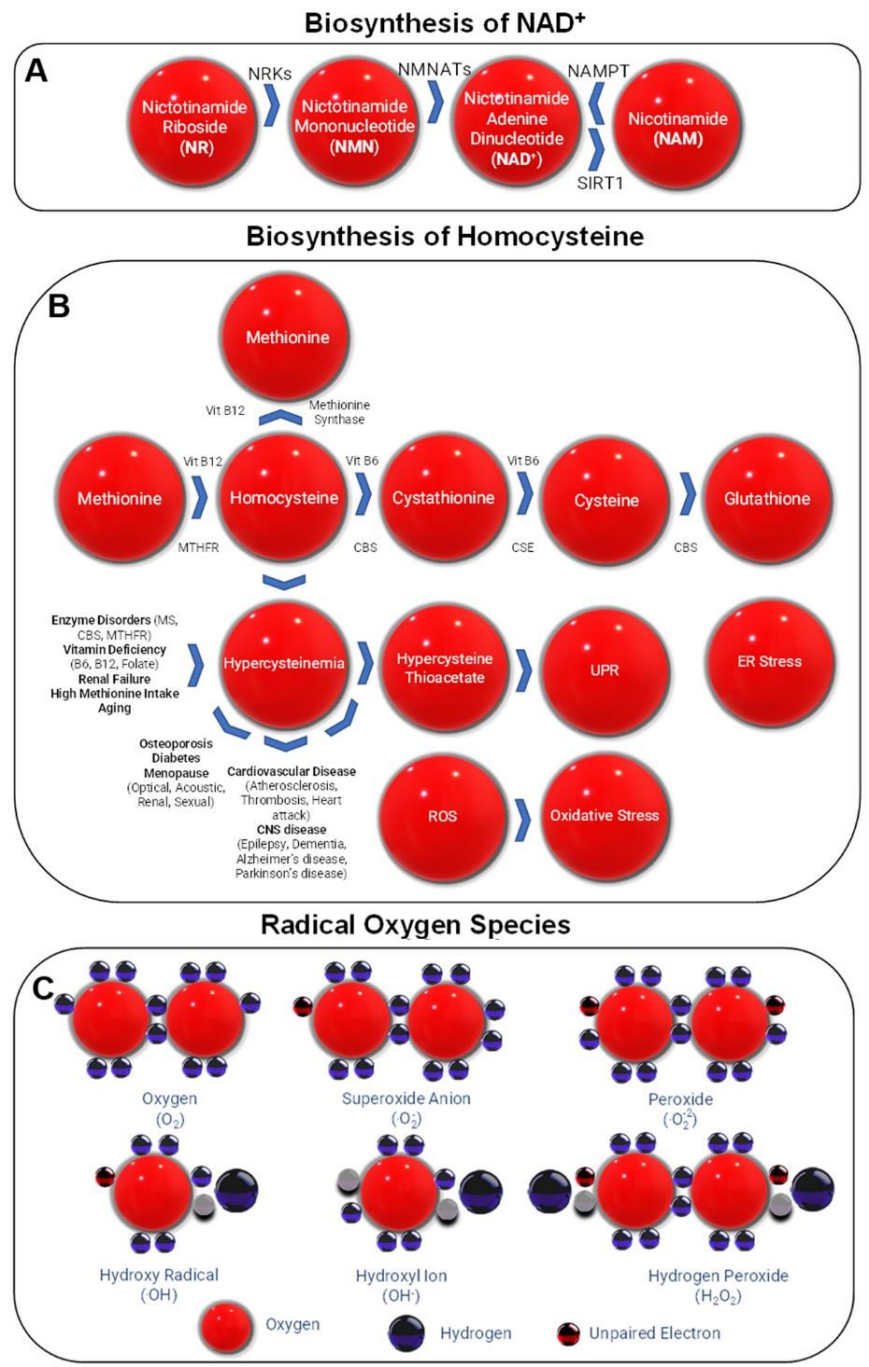
Biosynthesis of NAD+ (+ is superscripted) and homocysteine and radical oxygen species. (A) Biosynthetic pathways of NAD^+^, (B) Biosynthetic pathways of homocysteine, (C) Structure of Radical Oxygen Species (ROS).

NAD^+^ is a central and essential metabolite and an important co-factor in all living cells. For this reason, the NAD^+^ synthesis and degradation are exquisitely controlled. In mammals, NAD^+^ is synthesized from one or more of its major precursors including its principal substrate, nicotinamide (NAM), as well as tryptophan (Trp), nicotinic acid (NA), nicotinamide mononucleotide (NMN), and nicotinamide riboside (NR) (Fig. 5A). NMN is synthesized from nicotinamide, by the rate-limiting enzyme, nicotinamide phosphoribosyl transferase (Nampt), and 5′-phosphoribosyl-1-pyrophosphate (PRPP), or in the salvage pathway generated, by NR kinases (NRKs), from NR involving a phosphorylation reaction which allows it to be converted to NAD^+^ by NMN adenylyl transferases (NMNATs) [446].

NAD^+^ participates in numerous biological processes ranging from production of ATP via anaerobic glycolysis, OXPHOS, fatty acid β-oxidation, and tricarboxylic acid cycle metabolism (Krebs cycle). By virtue of engaging NAD^+^-dependent sirtuins, NAD^+^ regulates diverse cell signaling, gene expression profiles, and DNA damage repair [446]. NAD^+^ is essential to metabolism and induces expression of metabolic genes through its action on circadian clock [447]. NAD^+^ is required for the actions of poly (ADP-ribose) polymerase proteins (PARPs), namely PARP1 and PARP2 in sensing and responding to DNA damage which involves protein de-acetylation and poly-ADP-ribosylation (PARylation) [448-450]. In *C. elegans*, increase in levels of NAD^+^ by PARP inhibitors, activated the mitochondrial unfolded protein response (UPR^mt^), which is a mitochondrial proteostasis pathway, known to promote longevity and improved mitochondrial homeostasis through the activation of the sirtuin homolog, sir-2.1 [451-453]. The increase in NAD^+^ also activates the pro-longevity, FOXO transcription factor (daf-16), which engages antioxidant protection programs [454, 455].

It has been shown that aging leads to decreased levels of ATP and NAD^+^ in tissues and serum in *C. elegans*, to mice and humans [456-461] (Fig. 4A). Besides, aging and diseases, metabolic disorders, cancer, and neurodegenerative diseases also reduce intracellular NAD^+^ levels by reducing its synthesis or by increasing its consumption. Such a decrease reduces lifespan in *C elegans* whereas the genetic increase in NAD^+^ or restoration of its levels prevents metabolic changes that are associated with aging, leading to prolonged lifespan in *C. elegans* [455, 462]. The administration of NAD^+^ precursors, such as NAM, NMN or NR is an efficient way to substitute the lowered levels of NAD^+^ that occur with age [463, 464]. Long-term oral administration of NMN (up to 300 mg/kg), without inducing any toxicity, increases NAD^+^ in various peripheral tissues including brain tissues since NMN readily passes the BBB [465-467].

Aging is associated with disturbed circadian rhythms and sleep-wake cycles. Supplementation with NR erases such defects by inhibition of PER2 by its deacetylation at lysine 680 and its subsequent phosphorylation that regulates its entry into the nucleus [447]. Restoration of NAD^+^ to its youthful levels by supplementation with NR, reverses the age-related decrease in chromatin binding by BMAL1 which is required for oscillations of transcription, mitochondrial respiration and increased late evening activity [447].

In humans, administration of NR in 400 mg/kg produced dose-dependent increases in the blood levels of NAD^+^, and its metabolome, without inducing any adverse effect [468, 469]. NAM supplementation, protects the heart and brain against ischemia-induced damage, prevents age and high fat diet induced DNA damage, inhibits formation of glucoma, and restores diet-induced hepato-steatosis and glucagon storage in age-matched mice [470-473]. NR supplementation also reverses progressive muscle dysfunction and loss of muscle fiber integrity which occurs in old mice as a result of diminished NAD^+^ levels [468].

Administration of NMN maintains lipid and energy metabolism, increases insulin sensitivity, counteracts aberrant glucose tolerance, and immune functions, maintains bone density and affords protection in animals against age-associated functional decline [474, 475]. Similarly, NR improves glucose tolerance, and in models of diabetes and high-fat diet, improves metabolic function, prevents obesity, and reduces fat deposition [455, 476-479]. NMN supplementation reduces oxidative stress and inflammatory responses. NMN also maintains the population of neural stems and progenitor cells, prevents synaptic loss and protects aged mice against neuronal cell death, neurodegeneration and pathological damage in Alzheimer’s disease associated with Aβ, and the associated decline in the cognitive function [455, 488-490]. Similarly, NR supplementation delays the decline in cognitive function, improves learning and memory, maintains motor functions, and reduces neuronal cell death in animal models of AD and Parkinson's disease (PD) and prevents the development and progression of Aβ pathology in AD mice and *C. elegans* [484-487].

NMN supplementation provides protection against premature aging and extends lifespan and health-span [465, 488-490]. NAD^+^ dependent increase in health-span has been shown in normal aging mice, *Nampt*^+/-^ mice, β cell-specific *Sirt1*-overexpressing (BESTO) mice, hypomorphic BubR1 (a mitotic check-point kinase) mice, mice with age or diet-induced diabetes, and glucoma-prone mice [472-474, 491- 493]. NR prevents MuSC senescence in the mdx (C57BL/10ScSn-Dmd^mdx^/J) mouse model of muscular dystrophy, prevents the senescence of neural and melanocyte SCs, protects against aging and diseases and increases lifespan and health-span in many model systems [455, 476, 477, 494-496].

Thus, the available evidence shows that NAD^+^, is not only required in a large array of cellular activities, it has many rejuvenating features that promote a longer life-span and health-span. Replenishing reduced levels of NAD^+^ in aging by administration of NAM, NMN or NR restores homeostasis and provides protection against aging and age-related declines.

### Melatonin

Melatonin has a a wide range of health promoting effects including improving cell fitness and providing cell protection and survival [497]. Particularly, because of its nocturnal production by pineal gland which is controlled by light-dark cycles, melatonin participates in circadian oscillations [196] (Tables 4-5). The nightly increase in melatonin in humans starts at the 6^th^ to 8^th^ week of life, reaches its peak levels in 4^th^-7^th^ year of life. Moreover, its peak night levels progressively fall so that by the 7^th^ decade of life, there is no appreciable difference between the day and night-time levels [498-502] (Fig. 4C-D). Rhythmic secretion of melatonin at night defines biological night and endows this molecule to have rhythmic effect on a wide range of cell and body responses. Melatonin regulates sleep, circadian body temperature, and circadian rhythms [195, 196]. Light drives non-visual responses, including phase shifting the internal circadian clock, and increasing alertness, and heart rate. Melatonin also causes phase shifting of human circadian rhythms and protects against phase shifts which are artificially introduced by night-time exposure to light [195]. The therapeutic efficacy of melatonin hinges on increasing the dietary melatonin intake, sometimes using it at supra-physiological dosages [503]. The phase shift in the human circadian rhythms can be achieved by appropriate timing of administration of exogenous melatonin and light for the successful treatment of sleep disorders resulting from night shift work, circadian sleep disorders as well as jet lag induced by travel across substantially different time zones [195].

Melatonin increases mitochondrial respiratory efficiency, improves mitophagy, promotes mitochondrial biogenesis, and has been shown to protect mitochondria in the senescence-accelerated mouse strain, SAMP8 [504]. Melatonin enters mitochondria where it regulates the mitochondrial bioenergetic function. Melatonin increases the mitochondrial mass, mtDNA copy numbers and the energy supply, while decreasing the oxygen consumption. Melatonin protects mitochondria against DNA damage including those that occur as a result of intra-mitochondrial Aβ aggregates that accumulate in Alzheimer’s disease (AD) [505-508).

Melatonin provides resistance to oxidative stress by its antioxidant effect in countering generation of reactive oxygen species, and stimulation of antioxidant enzymes including superoxide dismutase, glutathione peroxidase, glutathione reductase, and catalase [501, 507]. Being both lipophilic and hydrophilic, melatonin readily passes cellular membranes and provides protection against ROS induced damage in various cell organelles [501]. Meltaonin prevents oxidatively induced telomere attrition, reduces damage to bioinformational molecules, proteins and lipids, and organelles, reduces the mitotically inactive-DNA damaged cells and associated SASP phenotype, prevents apoptosis, while increasing proliferation in leukocytes, stem cells and progenitor cells [508-510]. Melatonin also reduces inflammatory signaling and the development of senescence and immunoscenescence [508]. The pro-survival effects of melatonin have been attributed to its regulation of SIRT1 [511].

Senescence was delayed and lifespan was prolonged when the pineal glands of young mice were grafted into old animals [498]. Clinical trials show that melatonin prevents cell damage, in such conditions as sepsis, metabolic and neuro-degenerative diseases, cancer, inflammation, and aging [503]. Melatonin reduced lipid peroxidation, restored the locomoter activity, and increased lifespan in Transgenic Knockdown Parkin *Drosophila Melanogaster* exposed to paraquat [512]. Chronic administration of melatonin improved mitochondrial function and increased lifespan in senescence prone (SAMP8) mice [513].

Melatonin is also used in adjuvant therapy for age-related disorders including diabetes, neuro-degenerative diseases such as Alzheimer’s disease, glaucoma, macular degeneration, arterial hypertension, and insomnia associated with aging [514]. Together, the available evidene show that melatonin prevents age-related declines and increases lifespan and health-span [508].

### Alpha-ketoglutarate (AKG)

AKG, also referred to as 2-ketoglutaric acid, is an essential rate-determining intermediate in the Krebs cycle with a crucial role in cellular energy metabolism, protein, amino acid, mineral and lipid metabolism, and circadian patterns of lipid peroxidation [515-518]. AKG promotes higher protein and lower triacylglyceride levels in *Drosophila* [519). AKG also improves nitrogen balance, decreases protein catabolism and glutamine degradation, enhances protein synthesis, and for this reason, is used to reduce the protein catabolism during recovery from trauma, severe infection, burn, or surgery [520, 521]. AKG is a a mitochondrial redox sensor, that stabilizes redox homeostasis and affords protection against cyanogens, oxidative stress and ROS damage, and carbohydrate-induced cell death in *Saccharomyces cerevisiae* [522-527]. AKG modifies the metabolism by impacting levels of circulating hormones. Supplemention of the diet with AKG significantly increases circulating plasma levels of insulin, growth hormone (GH) and insulin like growth factor-1 (IGF-1) [528-529].

AKG maintains the pluripotency of the embryonic stem cells (ESC) and their *NANOG* expression and promotes the differentiation of primed human pluripotent cells [530-532]. Consistent with such a role in pluripotent cells, AKG significantly increases the number of inner cell mass (ICM) cells and promotes fetal growth [533].

AKG is neuroprotective and prevents induced motor dysfunction [534]. AKG significantly promotes proliferation and differentiation of mouse myoblasts, by impacting the glutamine metabolism, oxidative stress, and energy metabolism [535]. In the murine heart, AKG reduces pressure overload in chronic cardiac dysfunction and maintains elasticity of arterial wall in elderly mice [536, 537]. In middle aged mice, AKG causes adipose tissue rejuvenation by increasing the de-methylation and by increasing the expression of *Prdm16* promoter, promoting beige adipogenesis, and reducing gain in body weight and obesity [538].

AKG is involved in collagen metabolism by acting as a cofactor of prolyl-4-hydroxylase (P4H) that catalyzes the formation of 4-hydroxyproline, a crucial step in the formation of the collagen triple helix, and by facilitating collagen synthesis by increasing proline derived from glutamate [516]. AKG increases procollagen production in human dermal fibroblasts and decreases formation of wrinkles which are inducible by UVB exposures in skin of hairless mice [528, 529, 539, 540]. AKG increases BMP, is pro-osteogenic, supports bone development in rats and lambs, increases bone mass, overcomes age-related osteoprosis and protects bone loss due to hormone deficiency caused by ovariectomy [541-546].

AKG improves epithelial recovery induced by stress, protects the epithelial metabolic transition, and prevents dextran sulfate sodium (DSS) induced colitis [547, 548]. In pigs challenged with endotoxin, AKG improves the integrity of intestinal mucosa, alleviates oxidative stress and injury in intestinal mucosal cells, and maintains the absorption of nutrients [549].

AKG regulates major histone demethylases including ten-eleven translocation (TET) hydroxylases and the Jumonji C domain containing lysine demethylases, which catalyze the removal of repressive and age-related histone methylation marks (H3K9me3 and H3K27me3), and decreases their accmulation [532, 546, 550, 551]. By regulation of histone methylation, AKG reverses age-related osteoporosis [532]. AKG delays age-related decline in fertility in mammals, and partially prevents the age-related decline in locomotor activity and cold tolerance in *Drosophila* [519, 552]. AKG delays age-related phenotypes including hypercholestrolemia, increases lifespan in *Drosophila* and worms and reduces age induced morbidity in mice [553-556]. AKG reduced frailty scores by 46% in females and by 41% in males with higher frailty score correlating negatively with life expectancy [556]. The mechanism of action of AKG appears to depend on reducing NFκB mediated inflammatory pathway, hence, reducing the level of inflammatory cytokines. AKG also reduces ATP synthesis, by binding and inhibiting ATP synthase, and by inhibition of oxygen consumption. Its further actions are directed at activating autophagy and by decreasing the activity of TOR pathway [554, 556, 557]. The impact of AKG supplementation on increasing lifespan might also be related to its pleiotropic action on metabolism, reducing oxidative stress, and by endowing stress resistance. In a sense, the administration of AKG mimics calorie restriction, which is known to prolong life [558].

Together, the available evidence shows that AKG promotes cell fitness, health, and homeostasis, offers cell and organ protection, prevents, and protects cells against damage and prolongs health-span and lifespan in animals. For these reasons, AKG has rejuvenating features expected from a rejuvenin.

### Homocysteine

Homocysteine (Hcys) is a sulfur containing amino acid involved in one-carbon metabolism, that is derived from the essential amino acid, methionine via methionine cycle (Fig. 5B) Hcys is catabolized into cysteine through transsulfuration, a pathway that regulates cellular redox state by regulating levels of glutathione (GSH) and hydrogen sulfide (H_2_S) [559]. Hcys biosynthesis requires the participation of a host of enzymes including ATP-L-Methionine S-Adenosyltranferase (MAT), S-adenosylmethionine-dependent methyltransferases such as glycine N-methyltransferase, and S-adenosyl-homocysteine hydrolase. Homocysteine is converted by cystathionine β-synthase (CBS) to cystathionine which is coverted by cystathionine γ-lyase (CSE) to cysteine, ammonium and α-ketobutyrate. Homocysteine can be remethylated back to methionine by the participation of methionine synthase, methionine synthase reductase, vitamin B12, and 5-methyl-tetra-hydrofolate [560] (Fig. 5B).

**Table 7:** Cellular impact of chronic oxidative stress.

Item	Effect	Reference
*DNA*		
•Integrity	↓	269
•Global DNA methylation	↓	270
•Site specific DNA Methylation	↑↓	270
*Histone modifications*		
• Global acetylation	↓	271
•γH_2_AX	↓	272
Telomere		
•Telomere length	↓	273-276
•Telomere dysfunction	↓	277
Mitochondria		
•Function and energy balance	↓	278, 279
•ΔΨm	↓	278
•NAD^+^	↓↑	280
•TCA cycle	↓	282
•Glycolysis	↓	282
Deregulated Signaling		
•mTOR	↓	283
•NFκB	↓	284
Robustness of ER Stress Response		
•Unfolded protein response (UPR)	↓	285-286
Cell fitness		
•Proliferation, regeneration and number of population doublings	↓	273
•Cell signaling	↓↑	287
Damage Response		
•Oxidative-ROS	↓	288
•DNA (single & double strand breaks)	↓	272, 289
•Protein	↓	289
•Lipid	↓	289
•Cancer	↓	290-291
Repair Response		
•Cell and tissue repair	↓	292-294
•PARP activity	↓	295-296
Inflammatory Response		
•Inflammatory signaling	↓	297
Senescence		
•Hallmarks of senescence	↓	273, 288, 298, 299
•SASP	↓	289
Stem cells		
• Exhaustion	↓	299-300
Cell Death		
•Apoptosis	↓	301-302
•Necrosis	↓	288

↑ Increased ↓ Decreased

Hcys is a methyl donor, and its functions are important to the synthesis of methylated factors, folate metabolism, choline catabolism and in regulating methionine activity [561]. Hcys is also a potent oxidant and in plasma, by rapid auto-oxidation, Hcys generates stong oxidative products including superoxide and hydrogen peroxide [223]. The normal levels of Hcys in humans is between 5-15 μmol/l, and an increase above such levels causes hyperhomocysteinemia and homocystinuria, conditions that are associated with a host of cellular deficits and health issues [562, 563] (Fig. 4E, Table 6). Hcys levels in mild, moderate, and severe forms of hyperhomocysteinemia are, respectively, in the range of 15-30, 30-100 and above 100 μmol/l [252, 564-566]. Hyperhomocysteinemia leads to ROS induced oxidative stress, ER stress, angio- and neurotoxicity, and inhibition of cross-linking of collagen (Table 6) [567]. Excess Hcys increases the level of its highly oxidant metabolite, homocysteine thiolactone (HTL). HTL is highly reactive with ε-NH_2_ groups of the lysinic residues of proteins and changes their structure, activity, and function by protein homo-cysteinylation [568-570].

Mildest forms of hyperhomocysteinemia occur by deficiency of folate and vitamin B12, and in the advanced stages of renal disease [560, 571]. Interestingly, the folate deficiency which leads to hyperhomocysteinemia, coordinately dampens PER2 and vasopressin circadian rhythms and reduces responsiveness to photic resetting of the liver clock in mice [572]. Moreover, the pharmacological inhibition or knockdown of cystathionine β-synthase (CBS), which is involved in metabolism of Hcys and its deficiency, leads to hyperhomocysteinemia, significantly increases the amplitude of oscillations and baseline expression of Per2, suggesting an intimate link between the systemic levels of Hcys and circadian rhythms [573].

The impact of excess Hcys that leads to hyperhomocysteinemia and homocystinuria due to inborn errors in metabolism were first described in 1962 [562, 574]. Ten percent of the world population suffers from hyperhomocysteinemia due to reduced activity of the folate-metabolizing enzyme, MTHFR, which is caused by homozygous C677T polymorphism of MTHFR gene [561]. One of the most common forms of error in Hcys metabolism occurs due to the deficiency of CBS that prevents the conversion of Hcys to cystathionine and leads to deficient re-methylation of Hcys to methionine [560). Patients with hyperhomocysteinemia are at risk of developing multi-system disorders including vascular disease, vessel occlusion, cerebral ischemia, thrombo-embolic events such as pulmonary embolism, myocardial infarction, pancreatitis, seizures, osteoporosis, and mental retardation [562, 575]. Persistent hyperhomocysteinemia promotes endothelial cell dysfunction, inflammation, atherogenesis, and development of a thrombophilic profile [561].

Such findings have helped pave the way to the understanding of the role of Hcys in aging. Aging and age-related diseases lead to the aberrant metabolism of Hcys and cause hyperhomocysteinemia [559]. Aging leads to the progressive increase in the plasma levels of Hcys with values reaching over 10 μmole/L, particularly in males over age 50 [252, 565] (Figure 4E). The prevalence of hyperhomocysteinemia is 5-10% but might be as high as 30% in individuals over 65 with levels reaching 40 µmol/L [566, 576, 577]. Even in old but healthy individuals, hyperhomocysteinemia can contribute to the regional or widespread cortical atrophy associated with cognitive decline [578]. In fact, hyperhomocysteinemia, is a known risk factor for age-related diseases and a strong contributor and risk factor for cardiovascular diseases, atherosclerosis, Alzheimer’s disease, and other dementias as well as Parkinson's disease [561, 579]. The most severe forms of age-related hyperhomocysteinemia are associated with multi-system disorders that lead to the decline in organ function, peripheral vascular disease, atherogenesis, coronary artery disease, stroke, cognitive impairment, dementia, depression, osteoporosis and osteoporotic fractures, skeletal muscle myopathy, inflammatory syndromes, and rheumatism [252, 561] (Tables 5-6).

Together, the available evidence show that Hcys levels rise with aging and that such a rise leads to a host of age-related disorders

### Reactive Oxygen Species (ROS)

Denham Harman proposed oxidative stress as one of the most important drivers of aging [580, 581]. His hypothesis received further support when the superoxide dismutase (SOD), was identified as an enzyme that generates superoxide anions (O^-2^.) *in vivo* [582]. Identification of defense mechanisms against the oxidative stress and damage further boosted his original hypothesis. His idea was also compatible with the “rate of living” hypothesis, since it was shown that species with high metabolic rates, have faster aging rates and shortened lifespan, linking energy consumption to senescence and aging [583]. Ultimately, it was realized that higher respiration rates of mitochondria were the principal cause of production of reactive oxygen radicals, that were capable of inducing significant damage to cell constituents [584-587]. In mammalian cells, ROS are comprised of (.O_2_), Hydroxy Radical (.OH), Hydroxyl Ion (OH^-^), and Hydrogen Peroxide (H_2_O_2_). These species are generated within mitochondria by electron transport chain (ETC), through metabolism of peroxisomal fatty acid and xenobiotic compounds, mostly from plants, and by phagocytic and cytocidal release of hypochlorite as a “respiratory burst” for killing foreign pathogens [588] (Fig. 5C).

**Table 8:** Clinical impact of chronic oxidative stress.

Item	Effect	Reference
Barrier Inegrity		
•BBB dysfunction	↑	303-304
Bone		
•Bone mass	↓	305
ECM		
•ECM biosynthesis and organization	↓	306
•ECM degradation	↑	307
Energy and Metabolism		
•Insulin resistance	↑	308
•Appetite	↓	309
•Adipose tissue dysfunction	↑	310
GI tract		
•Epithelial injury	↑	311
Immune Response		
•Immune response (immunosenescence)	↓	312
•Inflammation	↑	312
Liver		
•Liver disease	↑	313, 314
Musculoskeletal System		
•Contractile function	↑	315
Nervous System		
•Memory	↓	316
•Cognition	↓	317
•Neurodegeneration	↑	289
Reproductive System		
•Fertility	↓	318
Skin		
•Pigmentation, wrinkles, aging	↑	319
Stem Cells and Tissue Regeneration		
•Stem cell function	↓	320
•Stem cell senescence	↓	321
Senescence		
•Senescence	↑	322-324
Urinary tract		
•Kideny pathology	↑	325
Disease risk		
•Aging	↑	323, 326, 327
•Disease	↑	289, 328
•Cancer	↑	328
Health-span and Life-span		
•Health-span	↓	323, 326
•Lifespan	↓	329

↑ Increased ↓ Decreased

ROS are ubiquitous second messengers and although, low levels of ROS are required for normal signaling and cell function, the rate of generation of ROS progressively increases with age, until oxidative stress prevails, inducing widespread damage to cellular and organ functions [288, 589]. Interestingly, humans or species with long lives, have better antioxidant mechanisms that protect them from such damages [590]. In addition, aging leads to the loss of enzymes such as catalase and glutathione peroxidase which break down hydrogen peroxide, further compounding the oxidative damage [591]. Sustained increase in ROS and lowered level of protection against ROS, ultimately, leads to activation of inflammatory signaling including those inducible by early growth response protein-1 (Egr-1), activator protein 1 (AP-1) and NF-κB [592]. The final outcome of such oxidative and inflammatory damages are oxidized molecules such as proteins and polyunsaturated fatty acids, declined cell function such as those related to mtDNA mutation, decreased mtDNA copy number, and compromised bioenergetics, senescence, fibrosis, and cell death. Ultimately, dysfunction appears in virtually every organ such as heart, liver, brain, and kidneys.

Together, the available evidence shows that, although certain levels of ROS are required for normal cell function, any increase above such levels causes chronic oxidative stress that manifest in aging and in age-related diseases (Tables 7-8).

### Discussion

Diverse lines of evidence show that youth and aging are plastic, requiring active maintenance. According to eco-centric hypothesis of aging, rejuvenation, and aging, are properties that emerge from the biologic impact of endogenous small molecules that circulate the blood, transit the basal laminae and BBB, enter the interstitium and ultimately pass the plasma membranes to initiate their rejuvenating or age inducing effect on cells. Most notably, parabiosis experiments show of existence of both rejuvenating (rejuvenin) and age inducing (geriatrin) factors. The labile nature and plasticity of youth and aging might be related to the diminishing levels of rejuvenins and escalating levels of geriatrins in the blood of aging organisms (Fig. 1). Consistent with such a hypothesis, whereas the centenerians do not suffer from a nocturnal loss in melatonin levels, all other aging individuals experience a flattened circadian profile for melatonin by the time they reach the 7^th^ decade of life [502]. Similarly, in contrast to falling levels of spermidine in old individuals, healthy nonagenarians and centenerians do not suffer from the loss of optimal spermidine concentration in blood and have levels that match those found in younger individuals [445].

There is increasing evidence that rejuvenation without undesirable effects can be achieved by parabiosis pairings of young and old organisms or by injection of plasma from young organisms. However, it is unlikely that similar approaches can lead to routine clinical treatment of aging in humans. The current methods of genetic reprogramming carry the risk of compromising the genetic integrity due to the possibility of insertional mutagenesis. Moreover, iPSCs or reactivation of telomerase have limited clinical use because of fear of tumorigenesis. Thus, although it is desirable to reset the epigenetic clock to an earlier time-point, it is not advisable to set it to zero. Clearly, cyclic genetic reprogramming is the leading method of choice since it was free of side effects [153]. Yet, this approach does not yield itself to clinical use of OKSM factors for rejuvenation. A method that has the likelihood of success for rejuvenation is the chemical induction of pluripotency. The early methods for chemically induced pluripotent stem cells or CiPSc still required introduction of *OCT4*, making such approaches to be clinically impractical [593-596]. However, using a combination of seven small-molecule compounds, Hou *et al* described successful reprogramming in mouse somatic cells, at a frequency of up to 0.2%, suggesting that reprogramming can be achieved merely by chemical rather than genetic means [597]. Thus, search for rejuvenins can include screening for chemical reprogramming, and/or factors that are competent to induce pluripotency and can reset the epigenetic clock to an earlier time-point (Table 9). Search for geriatrins can include identifying the small endogenous molecules that cause NF-κB or mTOR activation and a phenotype and molecular signature that are consistent with aging or those factors that can induce acceleration of epigenetic clock [183].

There is increasing evidence of the interplay between rejuvenins (NAD^+^, carnosine, spermidine, and melatonin), and geriatrins (Hcys). The idea that a geriatrin (Hcys) and a rejuvenin (NAD^+^), are metabolically linked, is clear from the fact that the NAD^+^ and Hcys biosynthesis are intertwined which directly links one-carbon metabolism and methylation balance to the levels of NAD^+^ [646]. Moreover, excess Hcys causes the depletion of NAD^+^ pools and significant fall in the NAD^+^ levels, likely due to an increase in the ROS since such a side effect could be prevented by radical scavengers [647]. Hyperhomocysteinemia is a risk factor in normal pregnancy. The administration of carnosine (100 mg/Kg) to pregnant rats that their pups were suffering from toxic effects of methionine induced prenatal hyper-homocysteinemia, restored the superoxide dismutase levels and prevented the cognitive decline and protected cerebellar neurons in the pubs from oxidative stress [648]. Carnosine prevented the oxidative stress in human erythrocytes that is induced by homocysteine and its by-product, homocysteic acid [649]. The suppression of liver S-adenosylmethionine which, leads to reduced spermidine levels, coordinately increased the plasma Hcys levels [650]. The key to the link between Hcys and melatonin, at least, to some extent, is related to the fact that melatonin synthesis requires methylation of acetyl-serotonin which is donated by S-adenosylmethionine and the by-product of this reaction, S-adenosylhomocysteine, is a substrate for homocysteine production [651]. In line with this, the levels of Hcys showed circadian rhythms and these oscillations are coordinated with the circadian melatonin release [652] (Fig. 4F). In addition, hyperhomocysteinemia, due to folate deficiency, is accompanied with decreased melatonin levels in the pituitary gland, and urine [651]. On the other hand, the removal of pineal gland, which reduces melatonin levels, leads to hyperhomocysteinuria [653]. Whereas pinealectomy did not change the circadian rhythms, it led to a substantial increase in mean plasma levels of Hcys [653]. Administration of melatonin to pinealectomized rats restored homocysteine to its normal levels and enhanced the antioxidant defense by raising the total glutathione levels [653]. Melatonin has been used to treat the adverse effects of a geriatrin (Hcys) in aged human population who suffer from hyperhomocysteinuria. The normal treatment for hyperhomocysteinuria is administration of folate (1 mg/day), vitamin B12 (400 µg/day) and vitamin B6 (10 mg/day), often, with mixed and not necessarily desirable results [654]. However, it was recently shown that melatonin reduces the oxidative stress induced by elevated levels of Hcys in nervous system and vessels [651]. Moreover, supplementation of melatonin to rats with chronic hyperhomocysteinemia, at a dose of 10 mg/kg, restored the decreased glutathione levels and significantly reduced the lipid peroxidation, and decline in cognition, learning and memory performance [655].

**Table 9:** The epigenetic and other conditions required for pluripotency.

Gene or epigenetic change	Pluripotent cells and ESC	Remark	Reference
Methylation and Methyltransferases			
Heterochromatin	Reduction of heterochromatin promotes reprogramming.	Heterochromatin represses gene expression.	598 599
Global DNA methylation (5mC)	Naïve pluripotency is associated with global DNA hypomethylation	DNA methylation silences somatic genes and chromatin remodeling during iPSC generation. DNA demethylation reactivates pluripotency genes, which are hypermethylated and silenced in somatic cells. Inhibitors of DNA methyltransferase improve the overall efficiency of the reprogramming.	600-603
H3K9me2 &3	A barrier to reprogramming. Reduction promotes reprogramming.	H3K9me2 &3 cover only 4% of the hESC genome, but over 10% of the human fibroblast genome. Dominant chromatin feature at the differentially bound regions (DBR)s, heterochromatic regions enriched for H3K9me3 in Fibroblasts.Repressive on Gene Expression. Megabase domains of H3K9me3 impair OSKM binding and reprogramming. Knockdown of relevant methyltransferases allows OSKM binding and enhances reprogramming. H3K9 methylation restricts late reprogramming events	598, 604-606
DNMT1	Impedes conversion of pluripotent to omnipotent state transition.	Totipotent zygote is essentially devoid of DNA methylation. DNA methylation is not required for pluripotency. Dnmt1 suppression leads to loss of DNA methylation on pluripotency-related genes and their expression is up-regulated, promoting pluripotency	607-609
EzH2	Presence is required for reprogramming	Catalyzes histone H3 methylation at lysine 27. Inhibition of PRC1 (Bmi1, Ring1) and PRC2 components (Ezh2, Eed, Suz12) significantly decreases reprogramming efficiency. EZH2 null blastocysts fail to generate ESC lines. The reprogramming capacity of Ezh2-depleted ESCs was severely impaired and several genes were either not induced (NANOG, REX1, TERT) or only partially induced (CRIPTO, OCT4). Long-term removal of Ezh2 (Δ/Δ) also resulted in compromised reprogramming. Ezh2 mediated H3K27me3 activity facilitates human pluripotent reprogramming and maintenance of bivalent chromatin domains in Pluripotent cells.	610-611
SUV39H1	Negative regulation or knockdown promotes pluripotency	Suv39h lysine methyltransferases (KMTs) generates H3K9me3 - a mark that is recognized by HP1 to maintain the heterochromatin	612
KAT7/HBO1	Dispensable for H4 acetylation		
Demethylation and demethylases			
5mhC	5hmC increases expression in ESCs and pluripotent cells.	5hmC promotes the demethylation of pluripotency genes during reprogramming. 5hmC enrichment is involved in the demethylation and reactivation of genes and regulator regions important to pluripotency. Promotes H3K36me2/3 demethylation in mouse embryonic fibroblasts in culture and during reprogramming.	598, 613, 614
KDM2A/JHDM1a	Dispensable for pluripotency maintenance in ESCs	The expression of pluripotency genes in Kdm2a^-/-^ESCs is downregulated and show defective lineage commitment upon differentiation. Regulates the expression of a subset of genes in ESCs. Promoters bound by Kdm2a are enriched in H3K4me3. Overexpression of potently enhances reprogramming, whereas knockdown impairs iPSC generation.	614-616
KDM2B/JHDM1b	AKG dependent protein involved in self renewal.	Kdm2b is a histone H3 Lys 36 dimethyl (H3K36me2)-specific demethylase, with the capacity to promote iPSC generation binds unmethylated CpG islands via a ZF-CXXC domain. Kdm2b is part of the PRC1.1 complex that mediates gene repression. Demethylases of histone H3K36 methylation. Increases gene expression and is required for generation of pluripotency. Overexpression of potently enhances reprogramming, whereas knockdown impairs iPSC generation.	598, 615, 616
Tet1, Tet2, TDG	Indispensable for maintaining pluripotency. Expression enhances reprogramming.	5-methylcytosine modifiers that cause 5mC to change to 5hydroxymC (5hmc), then 5-formylcytosine (5fC) to 5-carboxylcytosine (5caC). 5hmC, 5fc, and 5caC are enriched at regions bound by pluripotency factors. Oxidative demethylation is required to promote gene activation, to cause mesenchymal to epithelial transition and reprogramming of fibroblasts to pluripotency. Tet1 can replace Oct4 to generate fully pluripotent iPSCs. Tet2 mediated 5mC to 5hmC conversion on NANOG occurs in OKSM reprogramming. Loss of TDG leads to a significant decrease in pluripotency.	598, 613, 617-619
Acetylation and acetyl transferases			
H4ac	Critical regulator of pluripotency in embryonic stem cells		599, 620-623
H3K9ac	Increased H3K9ac predicts increased reprogramming to pluripotency. Enriched in mouse and human ESCs	H3K9ac is enriched at gene promoters and highly correlates with gene expression. H3K9ac level correlates with binding of transcription factors and (HDAC) inhibitors (HDACi such as valproic acid) that increase H3K9ac levels, restore reprogramming capacity. H3K9ac deposition enhances Sox2 binding. Most genes with high acetylation levels are bound by c-Myc. Increase in H3K9ac and decrease in H3K27me3 in promoters causes an active chromatin state.	599, 603, 622, 623
KAT1/HAT1	Coordinately increases with OCT4 and NANOG at 1 cell, 2 cell stage and blastocysts.	Acetylates histone H2A on lysine 5	624, 625
KAT6A and KAT6B	Acetylate both histone H3 and non-histone proteins.	KAT6A modulates Oct4 and Nanog binding to chromatin in ESC.	626, 627
KAT8/hMOF	Induces Generation of Pluripotent Stem Cells	MOF protein is dramatically upregulated following reprogramming/Kat8 plays key roles in embryonic stem cell core transcriptional network.	628, 629
p300/EP300	Proximal regulatory regions of pluripotency genes, such as OCT4 and Nanog shows H3K9ac	Recruited and directly interacts with Nanog to induce gene activation of Nanog-targeted genes in mESC.	630
Deacetylation and deacetylases			
HDAC1	Regulates and is essential to pluripotency.	HDAC1 occupies critical genes involved in maintaining self-renewal of ES and TS cells. Binds predominantly to pluripotency genes (OCT4, SOX2, NANOG, KLF4).	630, 632
SIRT1	Required for deacetylation of Oct4 and maintenance of naive pluripotency.	Oct4 is hyper-acetylated during the transition to pluripotency. SirT1-knockout reverses the induction of the primed pluripotency network.	633
SIRT6	Addition to Yamanaka factors improves reprogramming efficiency of HDFs	Highly expressed in pluripotent cells. Regulates the efficiency of mouse somatic reprogramming. Binds to the promoters of pluripotency genes. A H3K56 deacetylase controls embryonic stem cell fate via TET-mediated production of 5-hydroxymethylcytosine.	634-637
Others			
Lamin B1			638
FOXO3a	An essential regulator of pluripotency in hESC.	Contributes to reprogramming.	638, 639
NRF2/NFE2L2-KEAP1	Controls self renewal and pluripotency of hESC.	NRF2 inhibition directly disrupts self-renewal of hESCs and cellular reprogramming, and Nrf2 activation delays differentiation of hESCs.	640
cMyc	Prevents downregulation of pluripotent genes and plays crucial roles in generation of iPSC.	Enhances the binding of the other pluripotency factors to chromatin. cMyc expression leads to efficient induction of iPSC. cMyc alone can keep the mESC cells in pluripotent state.	614, 641-643
AKG	High level alters chromatin. Maintains pluripotency.	A cofactor for several chromatin-modifying enzymes, including histone demethylases and the Tet family of enzymes that are involved in DNA demethylation. Increased gene expression	598 644
HIRA	Dispensable for pluripotency in mESCs		645

ESC: embryonic stem cells. hESC: human embryonic stem cells, mESC: mouse embryonic stem cells, HDF: Human dermal fibroblast, iPSC: induced pluripotent stem cells, H4ac: Acetylated Histone 4, 5mC: 5 methyl cytosine, 5hmC: 5 hydroxy-methylcytosine

The key to the successful treatment of aging population is the parenteral rather than oral administration of rejuvenins since their absorption from gastrointestinal tract might be impeded by the degeneration of villi and decline in the number of nerve cells of the myenteric plexus in old individuals [656]. Moreover, the treatment should be tailored in such a way that the normal youthful circadian values of the rejuvenins and geriatrins are restored [446].

We presented the portraits of potential rejuvenins that show a wide range of rejuvenating effects as well as sample geriatrins whose effects simulate many of age-related declines and disorders. The age dependent decline in rejuvenins appear to underlie many features of aging which are further exacerbated by a coordinate increase in putative geriatrins. To this end, we truly hope that this primer can motivate those in the field to add their own rejuvenins and geriatrins to the rejuvenome and geriatrome atlases. The data and tools are publicly available at our Web Portal at www.bioscience.org/RGmoics. As shown by examples here, the rejuvenins and geriatrins might create interacting networks, akin to cellular molecular repertoires. We also hope that once the identity of all rejuvenins and geriatrins are revealed, the ensemble supplementation of lost rejuvenins alone, or in conjunction with strategies that suppress geriatrins, can lead to effective treatment of aging.
